# Comparative genomics and interactomics of polyadenylation factors for the prediction of new parasite targets: *Entamoeba histolytica* as a working model

**DOI:** 10.1042/BSR20221911

**Published:** 2023-02-09

**Authors:** Rodolfo Gamaliel Avila-Bonilla, Jorge Antonio Velazquez-Guzman, Eimy Itzel Reyes-Zepeda, Jorge Luis Gutierrez-Avila, César A Reyes-López, Alondra Cisneros-Sarabia, Emma Saavedra, Angel Lopéz-Sandoval, Esther Ramírez-Moreno, César López-Camarillo, Laurence A. Marchat

**Affiliations:** 1 Laboratorio de Biomedicina Molecular II, ENMH, Instituto Politécnico Nacional, Mexico City, Mexico; 2 Facultad de Ciencias, Universidad Autónoma del Estado de México. Carretera Toluca-Ixtlahuaca km 15.5 Cerrillo Piedras Blancas 50200 Toluca, Estado de México, Mexico; 3 Posgrado en Ciencias Químico-Biológicas; Escuela Nacional de Ciencias Biológicas, Instituto Politécnico Nacional. Mexico City, Mexico; 4 Laboratorio de Bioquímica Estructural, Instituto Politécnico Nacional, Escuela Nacional de Medicina y Homeopatía, Mexico City 07320, Mexico; 5 Departamento de Bioquímica, Instituto Nacional de Cardiología, Mexico City 14080, Mexico; 6 Posgrado en Ciencias Genómicas, Universidad Autónoma de la Ciudad de México (UACM), Mexico City, Mexico

**Keywords:** bioinformatics, mRNA processing, protein-protein interactions, protozoa, therapeutic markers

## Abstract

Protein–protein interactions (PPI) play a key role in predicting the function of a target protein and drug ability to affect an entire biological system. Prediction of PPI networks greatly contributes to determine a target protein and signal pathways related to its function. Polyadenylation of mRNA 3′-end is essential for gene expression regulation and several polyadenylation factors have been shown as valuable targets for controlling protozoan parasites that affect human health. Here, by using a computational strategy based on sequence-based prediction approaches, phylogenetic analyses, and computational prediction of PPI networks, we compared interactomes of polyadenylation factors in relevant protozoan parasites and the human host, to identify key proteins and define potential targets for pathogen control. Then, we used *Entamoeba histolytica* as a working model to validate our computational results. RT-qPCR assays confirmed the coordinated modulation of connected proteins in the PPI network and evidenced that silencing of the bottleneck protein EhCFIm25 affects the expression of interacting proteins. In addition, molecular dynamics simulations and docking approaches allowed to characterize the relationships between EhCFIm25 and Ehnopp34, two connected bottleneck proteins. Interestingly, the experimental identification of EhCFIm25 interactome confirmed the close relationships among proteins involved in gene expression regulation and evidenced new links with moonlight proteins in *E. histolytica*, suggesting a connection between RNA biology and metabolism as described in other organisms. Altogether, our results strengthened the relevance of comparative genomics and interactomics of polyadenylation factors for the prediction of new targets for the control of these human pathogens.

## Introduction

Gene expression regulation allows cells to adapt and survive to environmental stimuli by switching on or switching off specific protein coding genes. After gene transcription in the nucleus, the 5′-end of pre-mRNA is covered by the cap structure, introns are eliminated through splicing and 3′-end is cleaved and protected by a poly(A) tail. Then, mature mRNA is exported to the cytoplasm where it is translated to generate the corresponding protein. Particularly, mRNA polyadenylation is a relevant step since it facilitates nuclear export of mRNA, enhances mRNA stability, confers protection against exonucleases degradation and improves translation efficiency [1].

In human, mRNA polyadenylation requires the participation of more than 80 proteins, but a set of 14 proteins forms the four main multiprotein complexes. The cleavage and polyadenylation specificity factor (CPSF) recognizes the poly(A) signal (AAUAAA) through the WDR33 subunit, the cleavage stimulation factor (CstF) binds the U/GU-rich sequence, which enables the recruitment of the cleavage factors I and II (CFIm and CFIIm); then, the CPSF-73 endonuclease promotes 3′-end cleavage at the poly(A) site that is determined by the binding of the CFIm25 subunit to the UGUA sequence. Finally, the poly(A) polymerase (PAP) generates the poly(A) tail with the cooperation of the poly(A) binding protein (PABPN1). Symplekin, PP1, and RBBP6 also participate in mRNA polyadenylation. Additionally, the experimental demonstration of protein–protein interactions (PPI) between polyadenylation factors and proteins participating in transcription and other post-transcriptional steps evidenced the existence of functional and integrated PPI networks for gene expression regulation, and indicated that these functional machineries act in a coordinated way to ensure the correct synthesis, processing and nuclear export of transcripts, and therefore the preservation of a many diverse physiological processes [2–4]. Therefore, any troubles in polyA tail formation can have critical consequences. Thus, different human pathological conditions, such has cancer, congenital abnormalities, as well as cardiovascular, endocrine, hematological, immunological, musculoskeletal, and neurological diseases, have been associated with alterations in mRNA polyadenylation due to deletion or mutations in RNA sequences, and deregulation of selected polyadenylation factors expression [5].

The pandemic of COVID-19 abruptly remembered us the danger of viral infections, but infectious diseases caused by protozoan parasites also threaten human health, mainly in developing countries with low socioeconomic conditions and poor hygiene circumstances. Among them, malaria caused by *Plasmodium* spp, diarrhoeal diseases produced by *Entamoeba histolytica, Cryptosporidium parvum, Giardia* *lamblia*, and *Cyclospora cayetanis*, Trichomoniasis caused by *Trichomonas vaginalis*, infections by *Leishmania* and *Trypanosoma* species, represent the most prevalent parasitosis. Moreover, the opportunistic parasites, *Toxoplama gondii, Acanthamoeba castellanii*, and *Babesia microti*, produce severe diseases in patients with compromised immune systems. Despite the existence of drugs against these protozoan parasites, their control remains tricky because of side effects, drug resistance, and high cost; therefore, the identification of new drug targets is critical. Because of its relevance for gene expression, several groups have studied polyadenylation in selected protozoan pathogens. Particularly, our group identified the polyadenylation machinery of *E. histolytica* through computational analysis of the parasite genome sequence and showed that it has CPSF, CstF, CFIIm complexes, only the 25 kDa sunubit of CFIm, as well as PAP, PAB, Symplekin, PP1, and RBBP6 [6] and reported the functional characterization of several proteins [7–10]. Notably, we showed that EhCFIm25 silencing produces parasite death [11,12], which confirms that polyadenylation factors represent promising therapeutic targets such it has been described in *Trypanosoma, Toxoplasma*, and *Plasmodium* [13–17].

Systems biology is a computational approach to understand how a set of genes, proteins and even organisms, are connected to each other to fulfill a specific biological function. Notably, several *in silico* strategies have been developed for predicting PPI networks whose organization and topology can contribute to the identification of key protein for network function and potential drug targets [18]. To date, this computational and mathematical methodology has been poorly applied to study genomic data of human pathogens that affect human health [19–21]. However, the application of system biology is particularly important to understand the intricate relationships between pathogens and human host [22–24]. Notably, comparative system biology in parasites and the human host represents an effective strategy to understand how polyadenylation factors are connected to each other and other proteins to fulfill specific biological functions in both systems. It is also a valuable computational approach for a better understanding and identification of drug targets in pathogens. With the aim of obtaining a comprehensive view of the differences between relevant protozoan parasites and the human host, and therefore contributing to the discovery of relevant proteins for controlling these pathogens, here we performed a comparative analysis of predicted complex PPI network of polyadenylation machineries. Using *E. histolytica* as a working model, we realized a docking study to better understand the predicted interaction between two parasite bottleneck proteins. Finally, we performed some experimental assays in an attempt to validate our computational results. Altogether, our results allowed us to propose polyadenylation factors as potential biochemical targets for the control of these human pathogens.

## Methods

### Protein datasets and sequences analysis

Amino acid sequences of human polyadenylation factors were recovered in FASTA format from the UniProt database (https://www.uniprot.org/). Genes codifying for polyadenylation factors of *Plasmodium* spp, *Leishmania* spp, *Trypanosoma* spp, *Giardia lamblia, Trichomonas vaginalis, Toxoplasma gondii, Cyclospora cayatenesis, Cryptosporidium parvum, Babesia microti, Acanthamoeba castellani*, and *Entamoeba histolytica* were identified using the BLAST tool from the EuPathDB Bioinformatics Resource Center (https://veupathdb.org/veupathdb/app) with the corresponding parasite genome databases, and the amino acid sequence information of each human polyadenylation factor as a query, according to the following criteria: (i) at least 20% identity and 35% similarity to the query sequence, (ii) *E*-value lower than 0.001, and (iii) absence of stop codons in the coding sequence. The orthologous relationships of identified sequences with the human factors were confirmed by reciprocal BLAST using Expasy-SIB-BLAST (https://web.expasy.org/blast/). To explore phylogenetic estimations between human and parasite polyadenylation factors, we performed Multiple Sequence Alignment (MSA) using the SeaView Version 4, with the arbitrary maximum-likelihood tree building algorithm [25]. Guide tree estimation was obtained and visualized with the Smart Model Selection (SMS) to create an evolutionary tree [26].

### Building host and pathogens polyadenylation interactome

Sequences of human and parasite polyadenylation factors were used for predicting protein–protein interaction (PPI) networks from STRING database (https://string-db.org/) and interactomes were analyzed with the Network Analyser plugin of Cytoscape v3.8.2 (https://cytoscape.org/) using the most stringent criteria. Refinement steps were conducted to remove nonessential nodes and false-positive unreliable interaction, node and edge weight were computed for each protein and significantly lower node and edge weight were discarded [23]. To compare PPI networks between host and pathogens, proteins were clustered into two connected modules. The first module was assigned to PPI among polyadenylation machinery, and the second module corresponded to PPI related to other processes. Proteins having the highest number of interactions were considered as hubs, and proteins connecting both modules in the network were classified as bottlenecks [24]. The functional enrichment analysis was carried out using stringApp from Cytoscape v3.8.2; Gene Ontology (GO) terms, KEGG Pathways, STRING clusters, and Interior domains were used to determine the overrepresented molecular function and biological processes of parasites and human PPI networks. Overrepresented molecular function and biological processes were chosen with a FDR < 0.01.

### Analysis of RNA-seq datasets

RNA-seq data information of parasite polyadenylation factors were obtained from transcriptomic resources in VEuPathBD (https://veupathdb.org/veupathdb/app) using experimental datasets corresponding to *E. histolytica* trophozoites growing in normal conditions versus subjected to serum starvation for 24 h or replenished with serum for 2 h, following starvation [27], and virulent versus non-virulent trophozoites [28]. We also included data about polyadenylation factors of *Plasmodium vivax* growing in different microenvironments and temperatures [29], hypnozoites versus mixed cells [30] and sporozoites in different stages [31], as well as two wild-type strains of *Trypanosoma brucei* (MITat 1.2, clone 221a) and mutant strains [32]. Heatmap and hierarchical clustering were visualized using R statistical software packages.

### Molecular modeling and docking

The 3D structures of EhCFIm25 and EhNopp34 proteins (sequences C4M2T1 and C4LU58 from Uniprot, respectively) were generated using an approach of distance-based protein structure prediction by deep learning with the RAPTOR-X server [33] and homology modeling with the SWISS-MODEL server [34]. The 3D models were refined with the ReFOLD server [35] and their stereochemical quality was assessed by Verify3D [36] and PROCHECK [37] servers. The refined models of EhCFIm25 and EhNopp34 were submitted to molecular dynamics (MD) simulations through the GROMACS suite [38] version 5.1, using the OPLS all-atom force-field [39]. For this, monomeric structures were independently solvated in a dodecahedral box with its nearest edge 1.0 nm away from the protein, and the TIP3P explicit water model was used for all simulations. Sodium and chloride ions (for EhCFIm25 and EhNopp34 simulations, respectively) were added for system neutralization and all electrostatic interactions were calculated through the Particle Mesh Ewald (PME) approach. Energy minimization was performed using the steepest descent algorithm for 5000 steps. Then, a restrained MD simulation of 1000 ps was performed to allow the solvent to relax; the peptide atoms were harmonically restrained to their position in the model with a force constant of 1000 kJ/mol/nm^2^. All simulations were performed at 300 K and 1 atm pressure. The free MD run was carried out for 100 ns with the same pressure- and temperature-coupling constants as the restrained run. All steps of the simulations were performed using periodic boundary conditions. The stability and conformational changes of the trajectory for EhCFIm25 and EhNopp34 structures were characterized by analyzing the root mean square deviation (RMSD), that quantifies how much a structure diverges from another, and therefore may indicate the stability of the protein structure during simulation or may also reflect high flexibility of different regions of the protein structure, the root-mean-square-fluctuation (RMSF) that reveals which regions of the structure are the most mobile and the radius of gyration (*R*_g_), which indicates a measure of a protein compactness, all calculated by tools included in the GROMACS software. Finally, the protein–protein docking study was conducted with the LZerD webserver [40] using the average structures of EhCFIm25 and EhNopp34 obtained from the last 75 and 80 ns of the MD simulation, respectively, which are the times from which both simulations converged. Then, the dimeric structure with the highest frequency of members inside each cluster and the best score was used to perform a second docking. The best output structure was chosen considering the frequency of members inside each cluster and the best multi-score ranking from LZerD server.

### *Entamoeba histolytica* culture

*Entamoeba histolytica* (strain HMI:IMSS) trophozoites were grown in standard aerobic and axenic conditions in TYI-S-33 medium [41]. Parasites were also submitted to heat shock and serum depletion [42]. Briefly, 3 × 10^5^ trophozoites in exponential growth phase were transferred to 25 cm² cell culture flasks. Following their adherence to the surface, parasites were incubated at 42°C for 4 h before being harvesting. In another experiment, 3 × 10^5^ trophozoites were grown in 25 cm² cell culture flasks in TYI-S-33 medium without bovine serum at 37°C for 12 h. Trophozoites (3 × 10^5^) recently obtained from amebic liver abscess in hamsters were also used as virulent parasites [43]. Finally, trophozoites (5 × 10^4^) were soaked with *EhCFIm25*-dsRNA (100 μg/ml) for four days to silence *EhCFIm25* gene expression as described [11].

### RNA extraction and real-time qRT-PCR

Total RNA (1 µg) of *E. histolytica* trophozoites cultured under different conditions as described above was obtained by the TRIzol reagent (Invitrogen) according to manufacturer instructions and used to validate PPI networks predictions and RNA seq datasets analysis through Real-Time qRT-PCR. For this, cDNA was synthesized using the SuperScript III Reverse Transcriptase (Invitrogen) according to manufacturer’s instructions in a GeneQ Thermal Cycler (BIOER, Hangzhou, China). The qPCR assay was completed using the SensiFAST™ SYBR Hi-ROX Kit (Bioline) with specific primers for selected genes (Table 1). All reactions were performed in a StepOne real-time PCR system (Applied Biosystems) with the following steps: enzyme activation at 95°C for 2 min, denaturation at 95°C for 5 s, and annealing/extension at 60°C for 30 s (40 cycles). Experiments were performed by triplicate with three biological samples and the relative expression of mRNA was determined by the 2^−ΔΔCt^ method using data of the *EhRNAPII* gene for normalization [44].

**Table 1:** Oligonucleotides used in real-time qRT-PCR assays

Gene name (locus)	Forward primer	Reverse primer
Ehnopp34 (EHI_068680)	5′-ACCTTCCAAAAATTCTTGATGAACG-3′	5′-ACGTGCTACTACACAATCAGCA-3′
EhNSA2 (EHI_099760)	5′-GAGAAGGCAGGAAAGTGGGA-3′	5′-ACGCATAGCTGCAGGTCTAA-3′
EhCPSF1 (EHI_106110):	5′-ACACCTGATTGTCCACCTCA-3′	5′-TCCTGCAAAATGCCATGGTTC-3′
EhMyb (EHI_000550)	5′-CATTCCAGAAACGCGACCTG-3′	5′-TTCAGTGGCATAGGCTGTGT-3′
EhsnRNPF (EHI_060400)	5′-GCAAATCCATCTTTAGTTGCACCA-3′	5′-CAGGAATTTGTCCGGGTGGA-3′
EhCLP1 (EHI_008100)	5′-AGACGACTTCAACACCGAGC-3′	5′-TTTGAGATGCGGGTTGTCCA-3′
EhCFIm25 (EHI_077110)	5′-TGGAGAAGATGATCCTGTTGAAG-3′	5′-TCTTTGACTTGACTTACATGAACTG-3′
EhRBPP6 (EHI_014000)	5′- ACAACGACAATTACCACCAGGA-3′	5′-GTTGGGTCATCATCTGGAGCA-3′
EhPAP (EHI_012040):	5′-GTGC AGGAGTTGCTGATGAC-3′	5′-TGTGGTGATCGTTTTGATGGA-3′
EhRNAPII (EHI_056690)	5′-GATCCAACATATCCTAAAACAACA-3′	5′-TCAATTATTTTCTGACCCGTCTTC-3′

### Cross-linking immunoprecipitation (CLIP) assays

*Entamoeba histolytica* trophozoites (3 × 10^5^) harvested in exponential growth phase were transferred to 25 cm² cell culture flasks and grown at 37°C for 24 h. Then, parasites were exposed to irradiation in a 254 nm UV-transilluminator (BioDoc-It Imaging System (UVP)) for 30 min at 4°C [45] and cytoplasmic (CE) and nuclear (NE) extracts were obtained using the NE-PER Kit (Thermo Scientific™) according to manufacturer’s instructions. Proteins were quantified by the Pierce™ BCA Protein Assay Kit and their integrity was confirmed by SDS-PAGE analysis. The absence of cross contamination between NE and CE was assessed by Western blot experiments using anti-EhPC4 (1:1000 dilution) as a nuclear marker [46], anti-EhPSP (1: 1000 dilution) as a cytoplasmic marker [47], and anti-HsCFIm25 polyclonal antibodies (GeneTex, GTX115535) (1:1,500 dilution), followed by goat anti-rabbit IgG horseradish peroxidase secondary antibody (Invitrogen, A16096) (1:10,000 dilution), and proteins were revealed with the Immobilon Western Chemiluminescent HRP Substrate (MILLIPORE).

Immunoprecipitation (IP) assays were performed using Dynabeads protein A/G (Invitrogen) following manufacturer recommendations. NE (100 µg) were incubated during 2 h at 4°C with Rabbit IgG Isotype (10 µg) and magnetic beads (10 µl) (Invitrogen) to remove non-specific interactions. On the other hand, Dynabead magnetic beads (25 µl) were coupled with 10 µg of anti-HsCFIm25 polyclonal antibody (GeneTex, GTX115535) and anti-EhCFIm25 polyclonal antibody, previously produced and validated by our group [8] for 30 min at room temperature with1X PBS 0.02% Tween-20. Then, beads were washed three times with 1× PBS 0.02% Tween-20 and beads–antibodies complex was incubated with precleared NE (100 µg) for 2 h at 37°C followed by three washes with wash buffer (20 mM Tris, pH 7.5; 0.5M NaCl and 0.05% Tween 20). Finally, magnetic beads–antibodies–proteins complex was resuspended in elution buffer (0.1 M glycine, pH 2.0) for 2 min at room temperature to dissociate the complex; the tube was placed on the magnet and the supernatant containing eluted antibody and bound proteins were collected.

Proteins corresponding to eight independent experiments with each antibody were pooled (150 µg) and delivered to the Laboratorio Nacional de Servicios Experimentales (LaNSE), CINVESTAV (Mexico), for protein identification by mass spectrometry analysis LC-ESI-HDMSE as described [48,49]. Briefly, the proteomic parameters included: trypsin for protein digestion and one missed cleavage allowed; automatic peptide and fragment tolerance, minimum fragment ion matches per peptide, 2; minimum fragment ion matches per protein, 5; minimum peptide matches per protein, 1; and a false discovery rate (FDR) ≤ 4%. Raw files containing MS and MS/MS spectra were quantified by PROTEINLYNX GLOBAL SERVER (PLGS) v3.0.3 software against *E. histolytica* (Strain: ATCC 30459/ HM-1:IMSS, downloaded from UniProt, 7959 protein sequences, January 3, 2022) concatenated with reverse database and the protein identification was considered significant with the probability score of 95 and 99%. Mass spectrometry analysis was performed twice from the pool of eight IP samples to assess the reproducibility of the data, and only proteins identified in both replicates were considered. The mass spectrometry proteomic data have been deposited to the ProteomeXchange Consortium via the PRIDE [50] partner repository with the dataset identifier PXD033620.

### Statistical analysis

Data of gene expression were analyzed by Prism-GraphPad software using the paired Student’s *t*-test to compare stress, virulence and EhCFIm25 silencing conditions with standard growth condition as control. *P*<0.05 was considered as statistically significant.

## Results

### Comparative analysis of human and parasite polyadenylation machineries

Polyadenylation machineries have been poorly studied in protozoan parasites. Moreover, most polyadenylation factors have not been experimentally annotated (low-level of analysis) in genome databases. Here, we conducted a BLAST approach using human proteins as queries and identify potential orthologs in several protozoan pathogens that affect human health. As shown in Table 2, BLAST results indicated that *E. histolytica* and *A. castellani* have the largest set of polyadenylation factors with 15 proteins, followed by *T. vaginalis* and *L. mexicana* with 14 proteins, while *G. lamblia* has the smallest one, with only six proteins. All parasites conserve at least one subunit of the four main complexes, CPSF, CstF, CFIm y CFIIm (except for *G. lamblia* that lacks both CF complexes). Only *E. histolytica* and *T. vaginalis* have the six subunits of CPSF (CPSF1, CPSF2, CPSF3, CPSF4, FIP1, and WDR33), the other pathogens lack one to four of them, and WDR33 (11/13) and CPSF160 (10/13) are the three more conserved subunits across this set of parasites. Three polypeptides of CstF (CSTF1, CSTF2, and CSTF3) were identified in *A. castellani* and *Plasmodium s*pp, while *T. vaginalis, L. mexicana, T. cruzi, B. microti,* and *G. lamblia* seem to have only one subunit. Except for *G. lamblia*, all protozoa have the CFIm25 protein, and only *A. castellani* and *T. vaginalis* have the larger subunits of CFIm. Similarly, no apparent CLP1 and PCF11 (CFIIm) homologue were found in *G. lamblia*, and both subunits were only found in *E. histolytica, A. castellani* and *L. mexicana*. Interestingly, PAP that is fundamental for poly(A) tail synthesis is present in all parasites and PABP2 is only missing in *C. cayatenesis* and *G. lamblia*. Lastly, several accessory polyadenylation factors were also predicted in some parasites, but Symplekin (SYMPK) was only detected in *L. mexicana*. Additionally, we identified distinct genes for several polyadenylation factors in two other protozoan parasites; thus, two genes correspond to CLP1 in *T. vaginalis*, and to CPSF30 in *T. cruzi*. In contrast, the searches for PAPα, PAPβ, and PAPγ variants, as well as PP1α (PPP1CA), PP1β (PPP1CB) and PP1γ (PPP1CC) isoforms identified the same gene sequence in parasites (Supplementary Table S1).

**Table 2:** Comparative composition of polyadenylation machinery in *Homo sapiens* (Hs) and selected protozoan parasites. *Entamoeba histolytica* (Eh), Acanthamoeba castellani (Ac), *Trichomonas vaginalis* (Tv), *Leishmania mexicana* (Lm), *T. brucei brucei* (Tbb), *T. cruzi* (Tc), *Toxoplasma gondii* (Tg), *P. vivax* (Pv), *P. falciparum* (Pf), *P. berghei* (Pb), *Babesia microti* (Bm), *Cyclospora cayatenesis* (Cc), and *Giardia lamblia* (Gl)

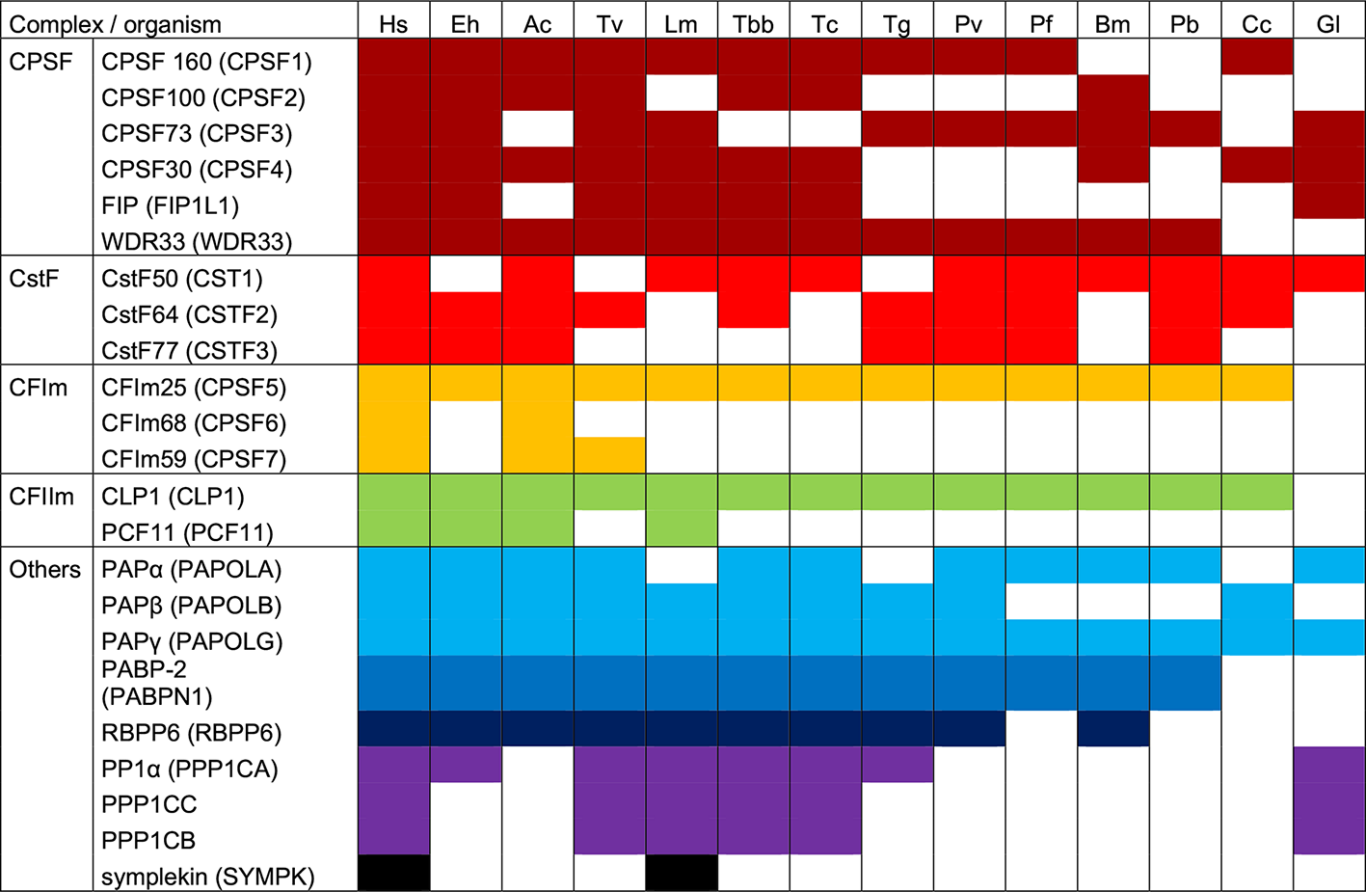

To better characterize the parasite polyadenylation machineries and assess their conservation, we next compared protein length and amino acid identity. Most polyadenylation factors have a similar size than the corresponding human proteins (Supplementary Figure S1). However, CPSF160 is significantly increased in size in *P. vivax, P. falciparum*, and *T. gondi*, as well as CstF77 in *P. vivax, P. falciparum, P. berghei*, and *T. gondi*, and PAP in A. *castellani*. In contrast, FIP1, WDR33, CFIm59, PCF11, and RBPP6 of all parasites correspond to ∼25–50% of the size of their human counterparts; the size of CstF64 is also reduced in *E. histolytica, T. vaginalis*, and *A. castellani*. In agreement with the BLAST search strategy, most parasite proteins share at least 20% identity with human counterparts (Supplementary Figure S2). Notably, PP1 isoforms were the most conserved factors with 70–90% identity in *T. gondi, L. mexicana, T. brucei brucei, T. cruzi*, and *G. lamblia*.

**Figure 1:**
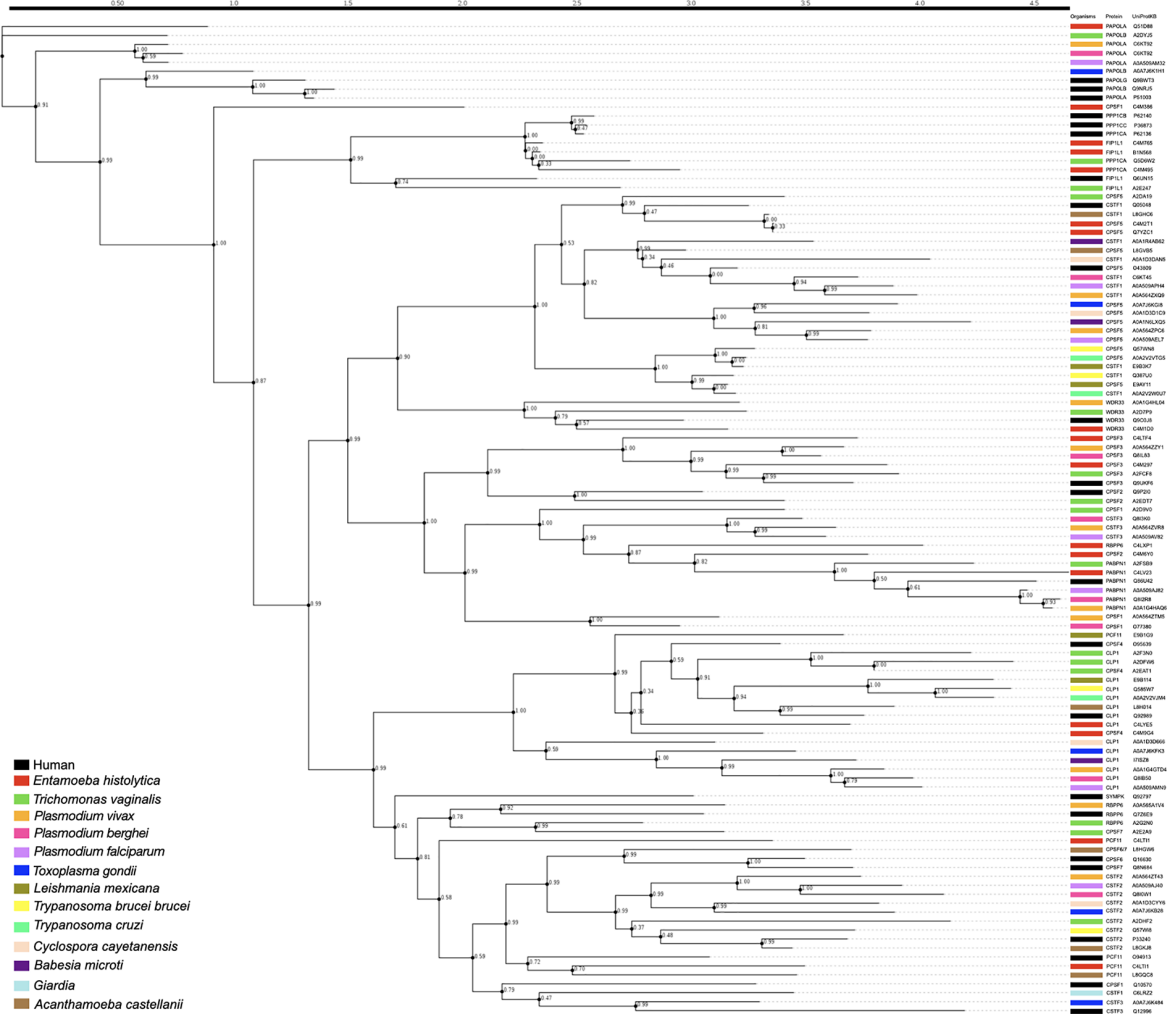
Evolutionary history of polyadenylation factors The phylogenetic tree was inferred by the arbitrary maximum-likelihood tree building algorithm and visualized with the Smart Model Selection (SMS). Each species is represented by a specific color, protein name and reference in the UniProt database are shown.

We also constructed the phylogenetic tree of parasite using human proteins as an external control group. The complete cladogram shown in Figure 1 revealed that some parasite proteins do not share a close relationship with their human homologue, since they present a polyphyletic origin in most pathogens. Notably, the poly(A) polymerases PAPOLA (*E. histolytica*) and PAPOLAB (*T. vaginalis*) share a polyphyletic relationship between them and are excluded from the main phylogenetic tree, suggesting that they evolved at a different time from the rest of the polyadenylation machineries. Interestingly, CFIm25 (CPSF1) of *E. histolytica*, shows a paraphyletic origin from the rest of the phylogenetic tree, suggesting that it does not share relationship with the human protein. Surprisingly, the human CstF50 (CSTF1) is closely related with CFIm25 (CPSF5) of *E. histolytica* and *T. vaginalis*, while the human CFIm25 (CPSF5) protein is related with CstF50 (CSTF1) of *Plasmodium* species, suggesting that these parasites proteins share similar functions. The human CPSF30 (CPSF4) protein is closely related to the CLP1 and CPSF30 (CPSF4) proteins in most parasites, as well the human proteins CFIm68 (CPSF6), CFIm59 (CPSF7), and CstF64 (CSTF2) with the pathogen proteins CstF64 (CSTF2), indicating that these proteins are efficient enough to remain throughout evolutionary history. In addition, the human SYMPK showed monophyletic grouping with CFIm68 (CPSF6), CFIm59 (CPSF7), PCF11, and CstF64 (CSTF2), revealing a positive selection of this protein on the history evolution of the polyadenylation. On the other hand, the human FIP1L1, PPP1CA, PPP1CB, PPP1CC, and WDR33 share monophyletic clustering with FIP1L1 and PPP1CA of *E. histolytica* and *T. vaginalis* and WDR33 of *Plasmodium vivax* and *T. vaginalis*, respectively. Other parasite proteins share monophyletic relationships with the homologue human protein, including CPSF73 (CPSF3), CPSF100 (CPSF2), CstF50 (CSTF1), and CFIm25 (CPSF5) of *Plasmodium, L. mexicana, T. brucei brucei, T. cruzi, G. lamblia, T. gondii, C. cayatenensis, B. microti*, and *A. castellani*.

### Human and parasite polyadenylations networks have different central hubs

**Figure 2:**
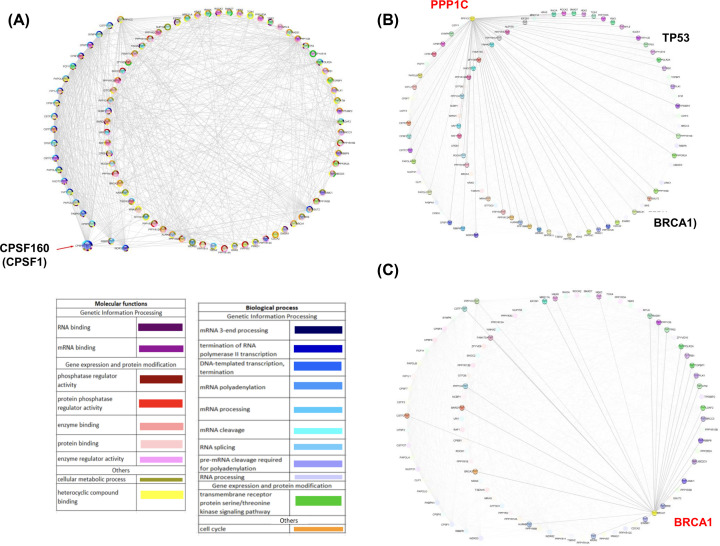
Analysis of polyadenylation factors interactions in human Data were obtained from STRING database and EuPathDB Bioinformatics Resource Center with the corresponding human genome database. PPI network was visualized using the Cytoscape tool, principal proteins corresponding to polyadenylation factors were represented in the external network. Internal interactions represent proteins associate with polyadenylation process. (**A**) Emphasis into the central hub protein (CPSF 160). (**B** and **C**) Emphasis into the bottleneck proteins PPP1C and BRCA1. GO terms including Molecular functions and biological process were carried out using KEGG Pathways, STRING clusters, and Interior domains. Proteins were marked with the corresponding GO term color.

The fact that some parasite polyadenylation factors have evolved in a different way than human proteins suggest that their function and their relationships with other proteins could be different. Moreover, the absence of factors may have an impact on protein function and their relationships with other proteins. Therefore, computational models of human and pathogens PPI networks were predicted using information available from STRING database to identify highly connected proteins (central hubs) for mRNA polyadenylation network. As shown in Figure 2A, the human network was clustered in two different PPI modules, specifically the polyadenylation module (external nodes) and the module corresponding to proteins that interact with polyadenylation factors (internal nodes). Interestingly, polyadenylation factors have relationship with proteins related to genetic information processing, as well as gene expression and protein modification. CPSF1 (CPSF160) represents the central hub of the network, establishing numerous connections (high degree of nodes) with polyadenylation factors and various proteins of the second module, suggesting that CPSF1 is likely to have a relevant role on the global network function via multiple interactions.

**Figure 3:**
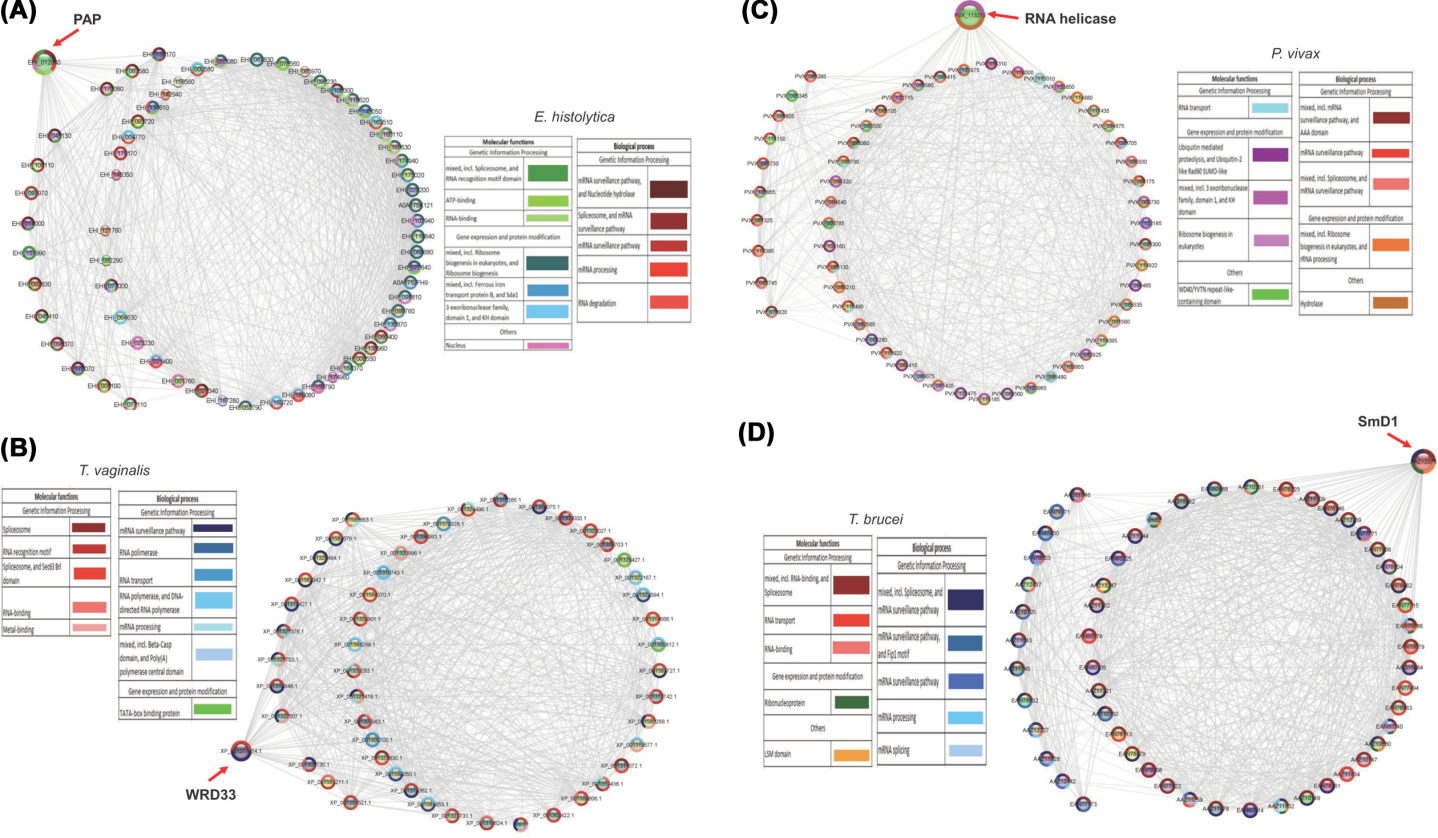
Prediction of polyadenylation factors interactions and hub proteins in selected protozoan pathogens Data were obtained from STRING database and EuPathDB Bioinformatics Resource Center with the corresponding parasite genome database. (**A**) *Entamoeba histolytica,* (**B**) *Trichomonas vaginalis*, (**C**) *P. vivax,* and (**D**) *Trypanosoma brucei*. The networks were visualized using the Cytoscape tool, principal proteins corresponding to polyadenylation factors were represented in the external network. Internal interactions represent proteins associate with polyadenylation process. Central hubs proteins are indicated with red arrows. GO terms including Molecular functions and biological process were carried out using KEGG Pathways, STRING clusters, and Interior domains. Proteins were marked with the corresponding GO term color.

Parasite networks contain the same two PPI modules; in addition to interactions within the polyadenylation module, polyadenylation factors also have relationships with proteins involved in gene expression regulation events. However, the central hubs of PPI networks are different in parasites and their human host. Specifically, some parasites networks display central hubs in polyadenylation proteins including PAP (EHI_012040; Q51D88) in *E. histolytica*, WDR33 (XP_001277014.1; A2D7P9) in *T. vaginalis*, and CPSF30 (XP_804858.1; Q4CRV4) in *T. cruzi*. In contrast, in other parasites networks, central hubs are proteins that interact with the polyadenylation module, such as the SSU ribosomal protein S7P (GSB_12981; V6TPX5) in *G. lamblia*, the small nuclear ribonucleoprotein SmD1 (AAZ12376; D6XKM9) in *T. brucei*, the nucleic acid binding protein (PBANKA_093980; A0A509AKK6), the RNA helicase proteins (PFF0100w; A0A024WC77 and PVX_113270; A5K1L9) in *P. berghei, P. falciparum*, and *P. vivax*, respectively, as well as the splicing protein Mago nashi (TGME49_067420; A0A125YHL8) in *T. gondii* (Figure 3 and Supplementary Figure S3).

### Interacting proteins exhibit a correlated gene expression

**Figure 4:**
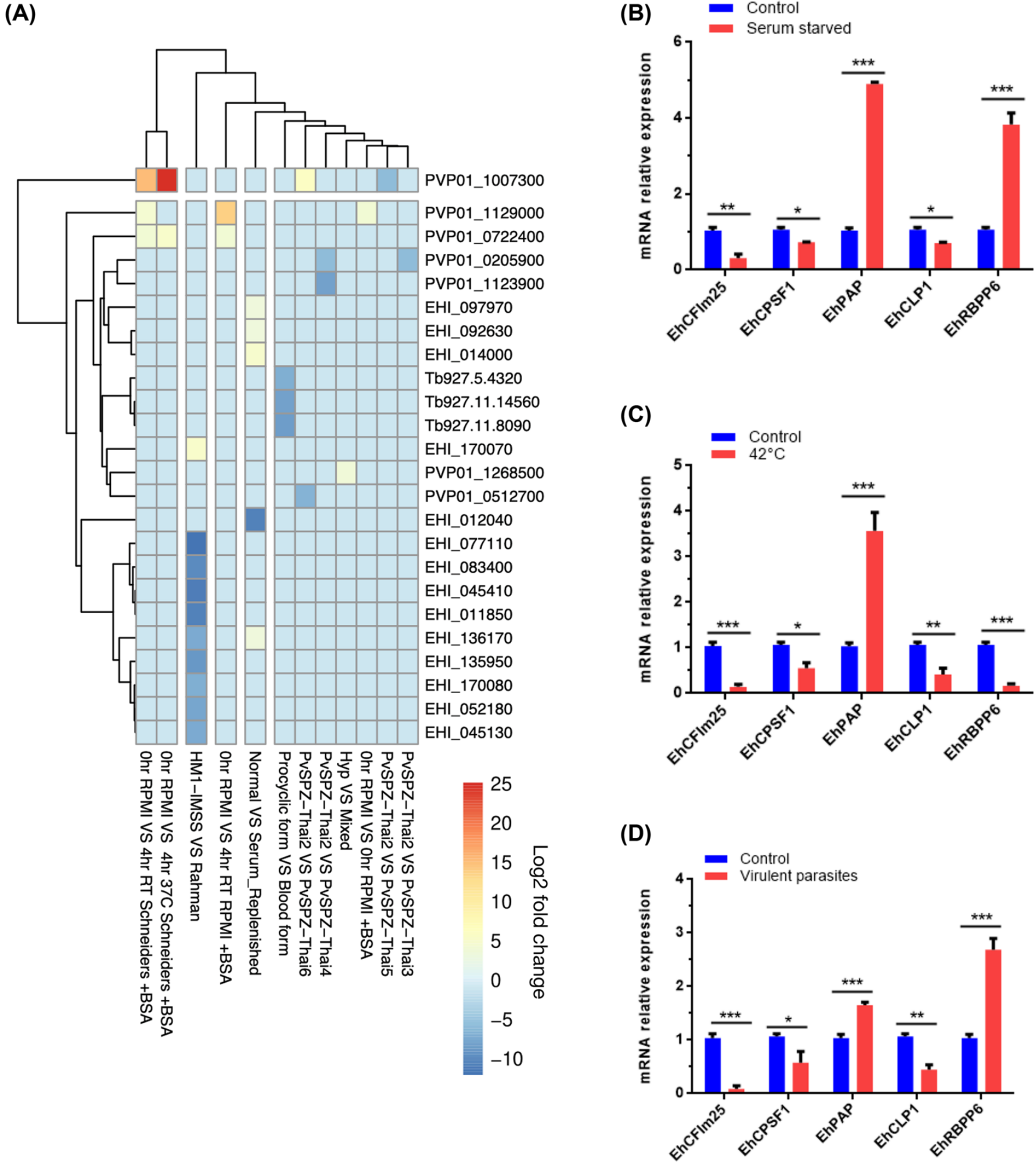
Analysis of polyadenylation factors expression in *E. histolytica* and other protozoan parasites (**A**) Heat map showing differential expression of polyadenylation factors growing in distinct conditions. Data were obtained from published RNA-seq experiments. (**B–D**) Real-time qRT-PCR for five *E. histolytica* genes selected from the heat map and PPI network. The EhRNAPII gene mRNA expression was used as normalization control for all qRT-PCR assays. Data corresponding to stress (serum starvation and heat shock) and virulent conditions were expressed as mean ± SD (*n*=3) and compared with the control condition using the paired Student’s *t*-test; *P<0.05, **P<0.01, and ***P<0.001.

To assess whether interacting proteins have a correlated expression, we retrieved the expression of several proteins in E. *histolytica* and other protozoa (*P. vivax* y *T. brucei*) PPI networks from RNAseq data in parasite databases. The heat map shown in Figure 4A evidenced the hierarchical clustering of modulated polyadenylation genes in different conditions. In *E. histolytica*, the expression of CPSF3 (CPSF73), WDR33, FIP, CSTF3 (CstF77), CPSF5 (CFIm25), and PCF11 genes is modulated in virulent trophozoites [28], while serum deprivation/replenishing affects CFSP3 (CPSF73), EhPAP, EhPABP, and EhRBPP6 expression [27]. The expression of CPSF1 (CPSF160), FIP, and PP1 was modified in the different life forms of *T. brucei* [30]. Similarly, the mRNA amount of CPSF3 (CPSF73), CSTF2 (CstF50), CSTF3 (CstF77), PABP, CPSF5 (CFIm25), CLP1, and PAP also varies in *P. vivax* according to growing conditions and life stage [29–31].

In the last two decades, our group has focused on the study of mRNA polyadenylation in *E. histolytica* [6–12]. Therefore, we used *E. histolytica* as a working model to perform several *in vitro* experiments to support computational predictions. First, we performed qRT-PCR assays to evaluate the expression of five genes, CPSF5 (CFIm25), CPSF1 (CPSF160), PAP, CLP1, and RBPP6, selected from the heat map and corresponding to proteins found in both modules of the PPI network (Figure 4B–D). As expected, all genes corresponding to interacting proteins were modulated in trophozoites grown in stress condition (serum-deprivation and heat shock) and in highly virulent parasites in comparison with controls. Interestingly, PAP was the most up-regulated gene in response to stress conditions, while CPSF5 (CFIm25), CPSF1 (CPSF160), and CLIP1 were reduced. The modulation of the RBPP6 gene depended on the stress condition. In virulent parasites, RBPP6 was the most overexpressed gene, followed by PAP, while the expression of other genes was reduced.

### *E. histolytica* polyadenylation network: central hub and bottleneck validation

**Figure 5:**
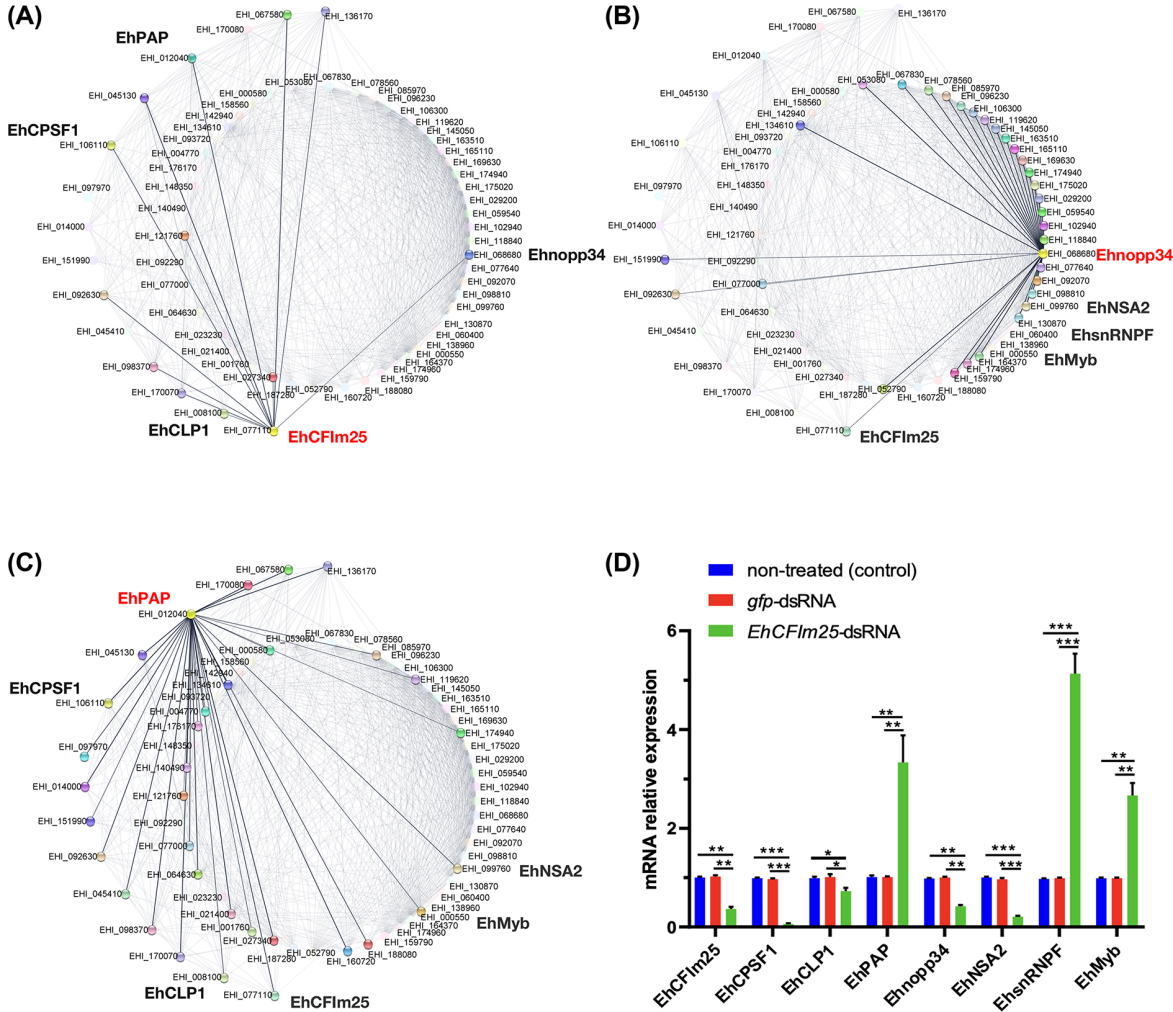
Protein-protein interactions network of polyadenylation factors in *E. histolytica* (**A**) Emphasis into the hub protein EhPAP (EHI_ 012040). (**B**) Emphasis into the bottleneck protein EhCFIm25 (EHI_077110). (**C**) Emphasis into the bottleneck protein Ehnopp34 (EHI_068680). Data were obtained from STRING database and EuPathDB Bioinformatics Resource Center with the corresponding parasite genome database. The networks were visualized using the Cytoscape tool, principal proteins corresponding to polyadenylation factors were represented in the external network. (**D**) Expression of relevant proteins identified in *E. histolytica* PPI network. Real-time qRT-PCR was performed for eight selected genes in EhCFIm25 silenced parasites. The EhRNAPII gene mRNA expression was determined and used as normalization control for all qRT-PCR assays. Data corresponding to the EhCFIm25-dsRNA and gfp-dsRNA conditions were expressed as mean ± SD (*n*=3) and compared with control cells using the paired Student’s *t*-test; *P<0.05, **P<0.01, and ***P<0.001.

As described above, PAP is a central hub in the PPI network of our working model *E. histolytica*. Interestingly, the node of the polyadenylation module with the highest betweenness is the polyadenylation factor EhCFIm25 (EHI-077110) that is known to be essential for parasite survival [11,12]; this bottleneck protein establishes interactions with nine proteins of the polyadenylation module and three proteins of the second module. Notably, EhCFIm25 interacts with Ehnopp34 (EHI_06680) that is the bottleneck protein in the second module (being connected to 28 nodes on this module and three of the polyadenylation module) and EhPAP (EHI_012040), the central hub protein in the interactome (Figure 5A–C). In contrast, the bottleneck protein that connects the polyadenylation module to the second module through its interactions with many proteins is PP1CC in the human PPI network. Notably, PP1CC interacts with two important proteins, BRCA1 and TP53, that act as a tumor suppressor proteins and interestingly, BRCA1 is a bottleneck protein in the second module (Figure 2B,C). The fact that human and parasite polyadenylation factors establish relationships with distinct proteins could be useful for target identification in *E. histolytica*.

Because of their high betweenness centrality in PPI networks, bottleneck proteins regulate the flow of signaling information and therefore, represent central points for communication in an interaction network [51]. To confirm the relevance of EhCFIm25 as a bottleneck protein, we evaluated the effect of *EhCFIm25* silencing on mRNA expression of relevant proteins identified in *E. histolytica* PPI network, particularly the hub protein EhPAP (EHI_012040), the bottleneck protein Ehnopp34 (EHI 068680), as well as EhCPSF1 (EHI_106110) and EhCLP1 (EHI-008100) that are connected to EhCFIm25 and EhPAP in the polyadenylation module, and EhNSA2 (EHI_099760), EhsnRNPF (EHI_060400) and EhMyb (EHI_000550) that are connected to Ehnopp34 in the other module (Figure 5D). Interestingly, the expression of these relevant was affected in trophozoites lacking EhCFIm25 in comparison with untreated cells, which confirms the importance of the bottleneck protein EhCFIm25 in regulating the flow of expression of these connected genes. Notably, EhCPSF1, EhCLIP1, Ehnopp34, and EhNSA2 were down-regulated, while EhPAP, EhsnRNPF, and EhMyb presented an increased expression. None of the genes were modulated in the *gfp*-dsRNA condition used as an additional control, confirming that the effects are due to *EhCFIm25* silencing by specific dsRNA as previously reported [11].

### EhCFIm25-Ehnopp43 interaction in *E. histolytica:* docking and model validation

**Figure 6:**
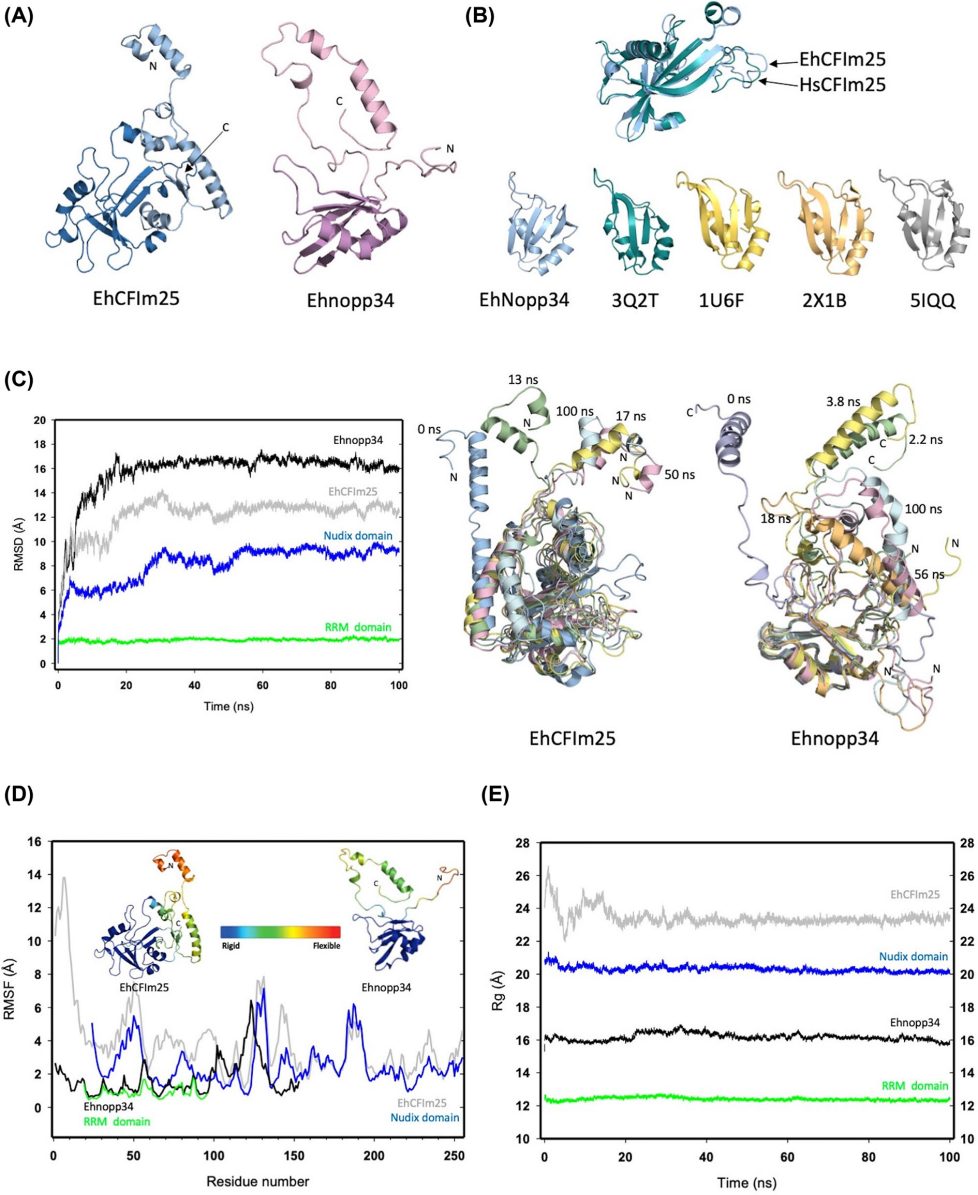
Modeling and molecular dynamics analysis of EhCFIm25 and Ehnopp34 (**A**) Cartoon representation of the refined models of EhCFIm25 and Ehnopp34. The Nudix domain of EhCFIm25 is shown in marine blue and the RRM domain of Ehnopp34 is highlighted in purple. (**B**) Superposition (upper panel) of the Nudix domains of EhCFIm25 (pale blue) and HsCFIm25 (green). The structures of the RRM domains of Ehnopp34, HsCFIm68 (3Q2T), UPB1 of *Trypanosoma cruzi* (1U6F), RNA15 of *Saccharomyces cerevisiae* (2X1B), and human RBM7 (5IQQ) are shown at the bottom. (**C**) On the left side, the RMSD graphs of the MD trajectory during the 100 ns of simulation are shown. The RMSD values of full-length EhCFIm25 (gray) and Ehnopp34 (black) were compared with those observed for the Nudix (blue) and RRM (green) domains of EhCFIm25 and Ehnopp34, respectively. On the right side, snapshots of structures obtained at different times of the MD showing the movement of the N-terminal segment of EhCFIm25 (N) and of the C-terminal segment of Ehnopp34 (**C**) through dynamics. (**D**) Graph of RMSF values through MD of EhCFIm25 (gray), Ehnopp34 (black), the Nudix domain of EhCFIm25 (blue) and the RRM domain of Ehnopp34 (green). The average structures of EhCFIm25 and Ehnopp34 are shown in the figure insert and are colored according to chain mobility during MD (rigid regions in blue and flexible regions in red). (**E**) *R*_g_ graph for EhCFIm25 (grey), Ehnopp34 (black), the Nudix domain of EhCFIm25 (blue) and the RRM domain of Ehnopp34 (green).

To assess the reliability of predicted PPI in *E. histolytica*, we investigated the interaction of bottleneck proteins in each module, EhCFIm25 and Ehnopp4. First, models with full sequence amino acids of each protein were generated. The RAPTOR-X server generated a full-length amino acid model of EhCFIm25 and a truncate EhNopp34 model (residues 19 to 151), while the SWISS-MODEL generated truncate models for EhCFIm25 (residues 29 to 236) and EhNopp34 (residues 1 to 108). The EhCFIm25 model generated by RAPTOR-X was selected for further analysis. To generate a complete sequence model of EhNopp34, the models obtained in RAPTOR-X and SWISS-MODEL were superposed (the RMSD of the Cα from residues 19 to 96 between both models was of 1.17 Å), and the atomic coordinates of amino acids 1 to 19 of the model obtained in SWISS-MODEL, were merged with the coordinates of the model obtained in RAPTOR-X using the coot software [52]. The refined models of EhCFIm25 and EhNopp34 showed 91% and 85% of residues in the most favored regions in the Ramachandran plot, 8.5% and 13.6% in additional allowed regions, and 0.4% and 0.7% in generously allowed regions, respectively, suggesting that the models obtained had suitable stereochemistry quality for MD and docking analyses. The EhCFIm25 model possesses the characteristic α/β/α Nudix fold of this protein family (Figure 6A). A superposition of Cα of the Nudix domain and Nudix box of EhCFIm25 (residues 92 to 230 and 137 to 161, respectively) and HsCFIm25 (residues 69 to 202 and 108 to 130, respectively) (PDB: 3Q2T) showed a RMSD of only 1.9 Å for the Nudix domain and 1.4 Å for the Nudix box, suggesting essentially the same folding (Figure 6B). We did not find any report about nopp34 crystallographic structure, but we observed that the predicted tridimensional structure of Ehnopp34 has the same domain of ∼90 amino acids folded into folded into β-1-α1-β2-β3-α2 fold than the RRM domain of HsCFIm68 present in the 3Q2T crystallographic structure of the human CFIm68/CFIm25/RNA complex. This folding similarity was confirmed when Cα of EhNopp34 RRM domain were superimposed with RRM domain of HsCFIm68 from the 3Q2T crystal complex (RMSD: 6 Å) or with other proteins containing RRM domains, such as the human RBM7 protein (RMSD: 1.9 Å; PDB: 5IQQ), the UPB1 of *Trypanosoma cruzi* (RMSD: 5.6 Å; PDB: 1U6F) or the RNA15 RRM domain of *Saccharomyces cerevisiae* (RMSD: 5.1 Å; PDB: 2X1B) (Figure 6B).

In the next step, we performed MD simulation of complete proteins and trajectories analysis of MD showed that the RMSD for Cα was around 13 Å for EhCFIm25, and 16.5 Å for EhNopp34, from 30 and 25 ns of simulation, respectively, that is the time in which each protein structure reached the convergence of the simulation. Interestingly, the analysis of the data extracted from trajectories corresponding to residues of the RRM and Nudix domains showed that the RMSD values for the RRM domain remained around 2 Å during the 100 ns of the simulation, while the RMSD profile of the Nudix domain appeared to be parallel to the RMSD curve of the full protein, although with lower values (approximately 4 Å). The main contributions to RMSD variations can be attributed to the N-terminal segment of EhCFIm25 (residues 1 to 23) and the N- and C-terminal segments of EhNopp34 (residues 1 to 18 and 97 to 155, respectively), judging by the structures extracted at different times of the DM (Figure 6C). Congruently, the central region of both proteins showed minor RMSF values, suggesting that the N- and C-terminal regions of EhCFIm25 and EhNopp34 have high flexibility in comparation to the core which presents a highly conserved folding (Figure 6D). Furthermore, the Rg showed a difference of approximately 3.3 Å between the full-length convergence structures and the folded core of both proteins, which reinforces the evidence that terminal segments of these proteins have high flexibility (Figure 6E).

**Figure 7:**
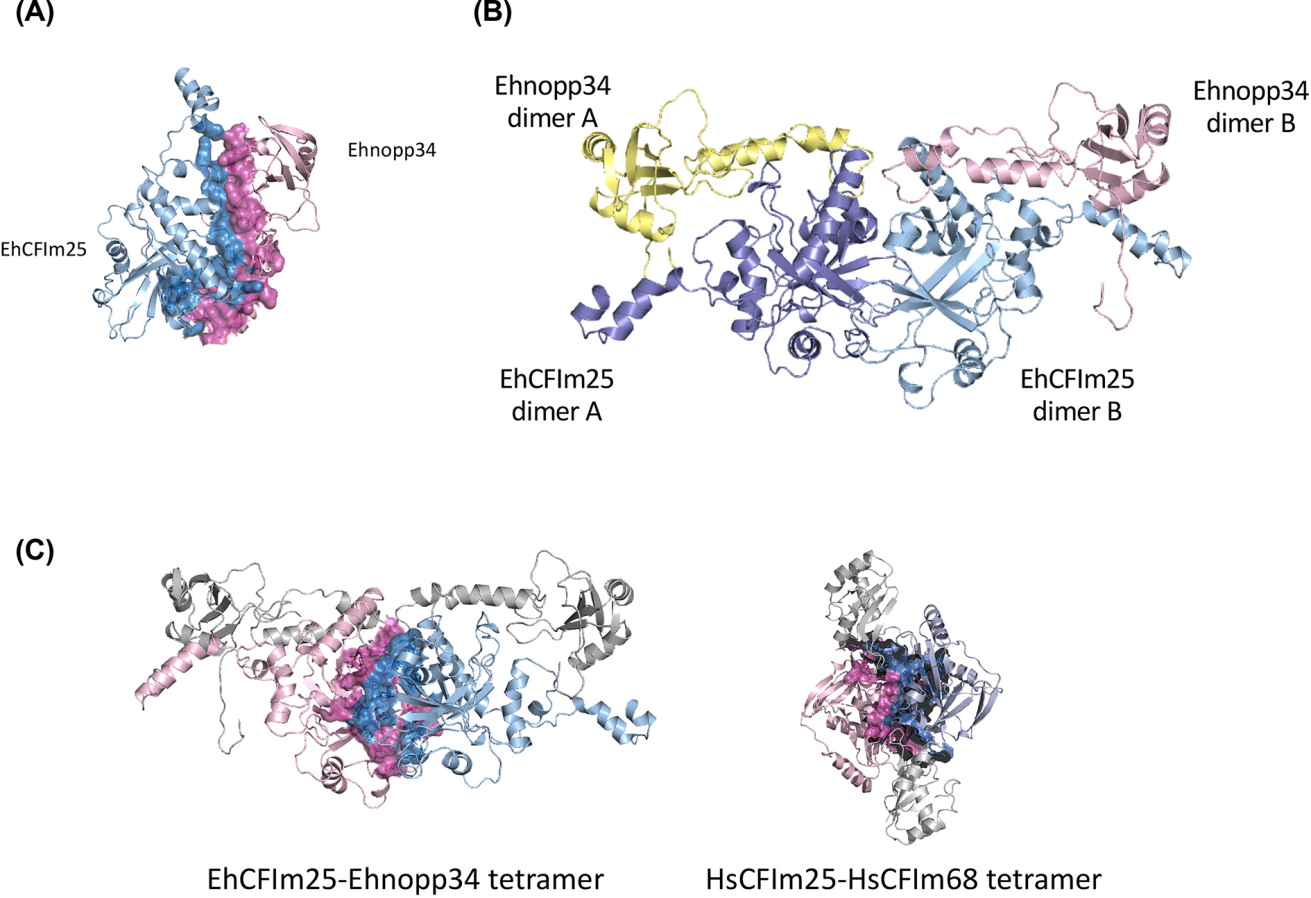
Docking analysis between EhCFIm25 and Ehnopp34 (**A**) Structure of the dimer formed by EhCFIm25 (light blue) and Ehnopp34 (light pink). The solvent-exposed surface of the residues that form the interface of EhCFIm25 (dark blue) and Ehnopp34 (pink) is highlighted. (**B**) Heterotetramer formed by a dimer of EhCFIm25 (in blue and violet) and two molecules of Ehnopp34 (in yellow and pink). (**C**) Comparison of the EhCFIm25–Ehnopp34 heterotetramer (on the left) and the HsCFIm25–HsCFIm68 (on the right). The surface of the CFIm25 dimer interfaces (in pink and blue for each CFIm25 monomer) is shown for each complex. The nopp34 and CFIm68 subunits of each complex are shown in gray.

Finally, to investigate the possible association between EhCFIm25 and EhNopp34, we used the molecular docking approach. With the LZerD server, a first dimeric molecule was obtained (one EhCFIm25 molecule and one EhNopp34 molecule), representative of a cluster of solutions and with the best score value. The interface between EhCFIm25 and EhNopp24 structures is formed by 36 amino acid residues of EhCFIm25 and 38 residues of EhNopp34 (Figure 7A). The binding energy of this dimer, estimated in the Prodigy server [53] was of −10 kcal mol^−1^, which is in the range of energies expected for protein–protein complexes. In mammals, CFIm25 has been reported to form homodimers [54] or heterotetramers with larger CFIm subunits [55]. Since EhCFIm25 is the only subunit found in *E. histolytica* and considering the similarity previously described between EhNopp34 and HsCFIm68 RRM domains, we decided to evaluate whether a heterodimer formed by EhCFIm25 and EhNopp34 could form a heterotetramer. The construction of the tetrameric molecule was performed from a dimeric molecule through a second docking, resulting in a tetrameric molecule in which the EhCFIm25 chains formed an interface, with an estimated binding energy of −6.9 kcal mol^−1^, while there were no interactions between EhNopp34 molecules (Figure 7B). The amino acids that formed the interface between both heterodimers are localized at the C-terminal of EhCFIm25, similar to the interface observed in the reported tetrameric structure of the HsCFIm25–HsCFIm68 complex (PDB 3Q2T) (Figure 7C) Interestingly, the binding energies of the human complex, estimated with the Prodigy server (−8.2 kcal mol^−1^ for the HsCFIm25–HsCFIm68 dimer and −13.4 kcal mol^−1^ for the HsCFIm25 dimer interface), agree with those observed in the *Entamoeba* complexes. The tetrameric model of EhCFIm25–EhNopp34 resembles the HsCFIm25–HsCFIm68 complex; however, the higher length of the complete model structures induces that the arrangement of each monomer of both EhCFIm25 and EhNopp34 is slightly different in comparison with the HsCFIm25–HsCFIm68 complex. Importantly, the RNA-binding sites of both proteins remain available in the obtained tetramer model (Figure 7C).

### Proteomic analysis of EhCFIm25 interactome

**Figure 8:**
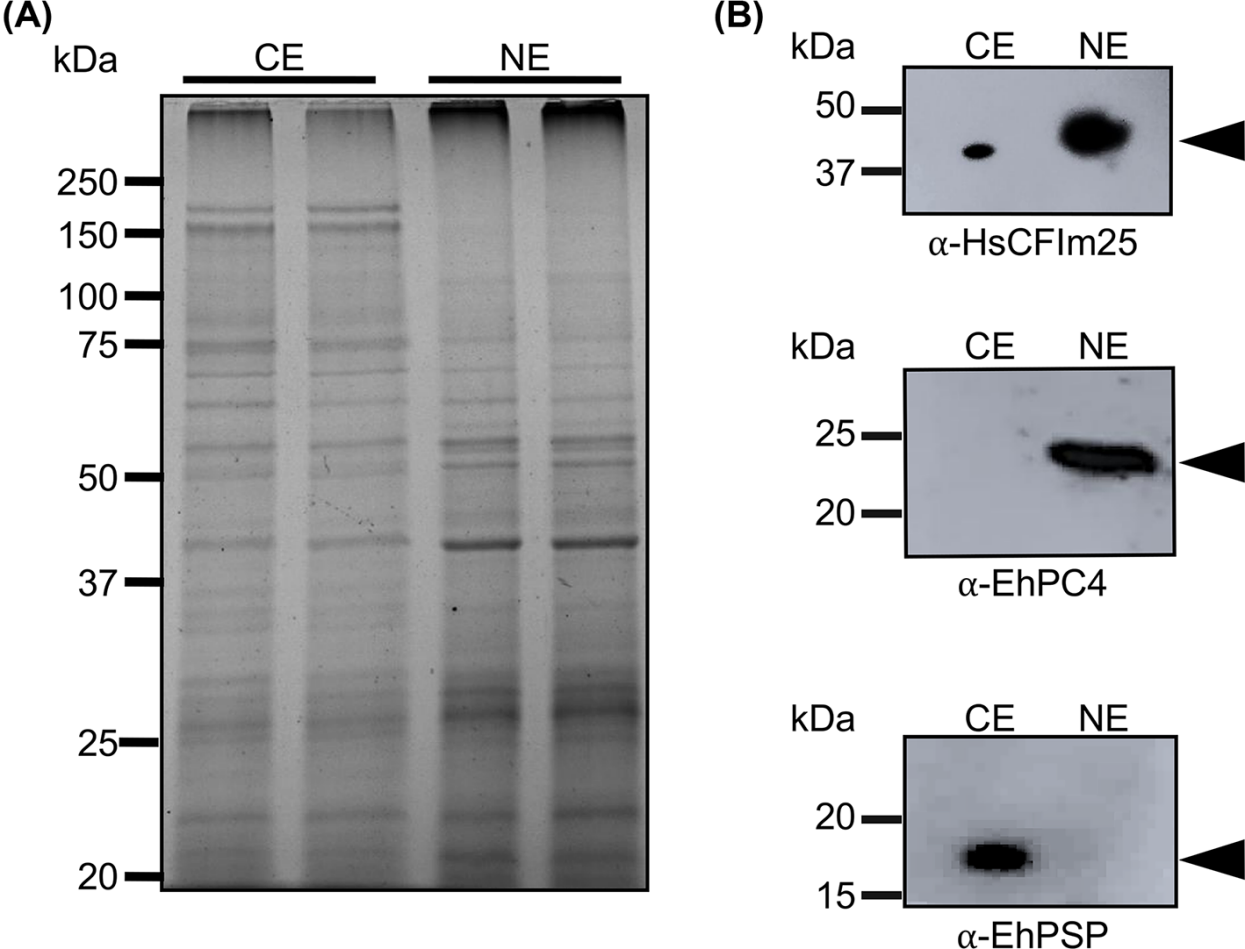
Analysis of cytoplasmic (CE) and nuclear (NE) extracts from *E. histolytica* trophozoites Protein extracts were fractioned by the NE-PER Kit (Thermo Scientific™) according to manufacturer instructions. (**A**) SDS-PAGE analysis that confirms protein integrity. (**B**) Western blot experiments using anti-HsCFIm25, anti-EhPC4, and anti-EhPSP antibodies that confirm the recognition of parasite CFIm25 protein by heterologous antibody and the absence of cross contamination between NE and CE.

In an attempt to experimentally identify EhCFIm25 interactome, we conducted a proteomic analysis of *E. histolytica* nuclear proteins that were immunoprecipitated by two different anti-CFIm25 antibodies. The quality of NE and CE was previously assessed by SDS-PAGE since migration profiles displayed clear differences (Figure 8A). Additionally, in Western blot assays, the nuclear marker EhPC4 was only detected in NE, while the cytosolic marker EhPSP was only found in CE, which indicates the clear separation between NE and CE, and a good enrichment in nuclear proteins. The EhCFIm25 protein was mainly detected in NE, but also in CE as previously described [8]. Moreover, results confirmed the recognition of parasite CFIm25 protein by heterologous antibody, confirming that it can be used in CLIP assays (Figure 8B).

As described above, two distinct antibodies were used for CLIP assays followed by protein identification by mass spectrometry analysis LC-ESI-HDMSE. Detailed information about protein identification is shown in Table 3. Although both strategies allowed the identification of distinct EhCFIm25 interacting proteins, some features are conserved, such as the presence of (1) proteins of the predicted interactome of polyadenylation factors shown in Figure 3, particularly the WD repeat protein and ribosomal proteins in the second module of the PPI network; (2) proteins assigned to nuclear functions (S1 RNA binding domain-containing protein, elongation factor 1-α, NEDD4-binding protein 2); and (3) proteins related to metabolism pathways that typically take place in the cytoplasm, namely glyceraldehyde-3-phosphate dehydrogenase and pyruvate phosphate dikinase that contribute to energy generation [56], as well as peroxiredoxin and thioredoxin that participate in the thiol-dependent redox metabolism and the ubiquitous oxidoreductase system with antioxidant and redox regulatory roles, respectively [57].

**Table 3:** EhCFIm25 interactome

Antibody	Protein ID	Protein name	Peptides seq	Protein sequence coverage (%)	*e*-value	Function
Anti-HsCFIm25	EHI_145840	Peroxiredoxin	● AIQFSDEHGAVCPLNWKPGKDTIEPTPDGIK ● APAYCPCGSIK ● YIQMNDDGIGR ● EIDINEYR ● STEETIR ● FINTFEK ● YIQMNDDGIGR ● INTFEK	32.19	1.688E-01	Cell redox homeostasis
	EHI_084260	Cys peroxiredoxin_ putative	● AIQFSDEHGAVCPLNWKPGKDTIEPTPDGIK ● APAYCPCGSIK ● EIDINEYR ● FINTFEK	26.27	2.2635E-01	Cell redox homeostasis
	EHI_138320	WD repeat protein	● GMNPAHVYCVALSDDGK ● WWFGVENNVSDYASLIIHDILPK ● MTQQEDTILCISINEQK ● DTFIALENNIDVYCEGK	21.15	5.813E-01	Autophagy of nucleus
	EHI_170420	Thioredoxin_ putative	● GNENIEFEGPR	9.24	1.7566E-01	Response to protein folding stress
	EHI_167320	Glyceraldehyde-3- phosphate dehydrogenase	● VGTPDVSCVDLTCR ● CACANIIPASTGAAK ● TVDGPSGKDWR	8.68	1.7591E-01	Glycolysis and Gluconeogenesis
Anti-EhCFIm25	EHI_077270	S1 RNA binding domain-containing protein	● DVTIHVAPGELLDNTSYHGKDFSR ● HENTTMEEEEEIEEGK ● IPFPTGYSLNPKDK ● IGYIHYCDISNVFNPFPR ● GITGR ● NNSIYLTHK ● INSGDIPIEDLQSVFKK ● IEGVVSYTNESISYLNLGK ● HDVWNIYIDMEKEVGDVGVIR ● CVIVNITSEGLFLR ● GNTMKCIVIGVDK ● NNYQTKNGNVMK ● LGDIIPLSVISPTTKENR ● DIKENIVTK	24.46	1.6987E-01	rRNA biogenesis protein in nucleolus
	EHI_040800	60S ribosomal protein L15	● FFEVILVDPFNAAIR ● VLNSYFIGQDSTYR ● VKSLQAIAEQR ● VLNSYFIGQDSTYR	19.51	3.8852E-01	Structural constituent of ribosome
	EHI_045090	Pyruvate phosphate dikinase, putative	● GAGLCTMTKIGLPVPQGFVITTEMCK ● VFGGEENPLLVSVR ● GAGLCTMTK ● VYAFEDGDGTNK	16.26	2.719E-02	Pyruvate, phosphate dikinase activity
	EHI_146560	Ribosomal protein S24_ putative	● TSGFALIYDTLSALK	10.71	2.3713E-01	Structural constituent of ribosome
	EHI_052400	Elongation factor 1-alpha 1	● THINIVVIGHVDSGK ● FEELLSK ● YYFTIIDAPGHR ● STTTGHLIYK ● QERYEEIK	8.37	2.9991E-01	Nucleocytoplasmic transport
	EHI_158100	NEDD4-binding protein 2	● ATIAIFEEVNIYMVDEDAIDLHGLQIDGALDMVK ● ELTWPIEDSIIYKK ● SIVKVQCGMGHHNTVGFSK	6.90	6.3809E-02	Transcription corepressor

## Discussion

It has been demonstrated that altering mRNA polyadenylation represents a valuable strategy for protozoan parasite control [11–17], but more information about the relationship between parasite factors and the human counterparts is required to better identify specific parasite proteins as drug targets. The present work combining computational methods and experimental assays was centered around two major research questions in relation to biology system studies: (1) do polyadenylation factors conserve the same relationships and protein connections in human and parasites? and (2) do polyadenylation factors and interacting proteins share a correlated expression?, whose answers allowed us to propose potential targets against parasites that still affect human health worldwide at the beginning of the XXI century.

Complementing the description of some polyadenylation factors in several protozoan parasites [6,58–60], our protein sequence similarity searches for polyadenylation factors in parasites suggested that polyadenylation machineries present a large variability among protozoa. Interesting, *E. histolytica* and *A. castellani* conserve 15 of the 23 main and associated polyadenylation proteins described in human, while *G. lamblia* only has six, as we previously reported [6]. Importantly, the conservation of CPSF160, CFSP73, CFSP30, WDR33, CstF50, CstF64, CFIm25, CLP1, PAP, PABP, and RBBP6 in almost all parasites suggest these proteins may have fundamental roles in mRNA 3′ end formation. Notably, WDR33 recognizes the polyA signal in human cells [61], CFIm25 defines the cleavage site [62,63], CstF73 performs RNA cleavage [64] and PAP catalyzes the poly tail elongation [65]. The absence of CPSF73 in *A. castellani, T. brucei brucei, T. cruzi* and *C. cayetanensis* (and other important proteins in *G. lamblia* that has the smallest machinery) suggests that other yet unknown proteins may have independently evolved to perform the corresponding functions in parasites. Importantly, the fact that the *E. histolytica* CFIm25 is evolutionary related to the human CstF50 suggests that it may be able to functionally replace the missing CstF50 in the parasite, making it a very relevant protein in the polyadenylation process. This assumption agrees with our previous reports showing that the absence of EhCFIm25 produces parasite death [11,12].

Besides amino acids sequence similarity, the comparison of amino acids number is another way to characterize the conservation of homologous proteins [66]. Interestingly, there are some parasite proteins that do not follow the general rule of protein length increase throughout evolution. For example, the size of CPSF160, CstF77 and PAP is increased in several parasites, in comparison to their human homologues, which adds another piece to the evolution of polyadenylation factors. Phylogenetic analyses also provide valuable data to understand the behavior of a protein, in comparison with other biological models studied, such the human polyadenylation machinery. Our analyses generally support the evolutionary relationships among parasite and human polyadenylation factors. Despite their sequence similarity, some proteins, do no share a common origin with human homologues, which suggests that their formation has been most likely mediated by different evolutionary events. It is possible that these differences could affect protein function and PPI in each system. Congruently, these differences have an impact on the organization and topology of predicted PPI networks although their sequence similarity suggests that homologous proteins conserve the same functions in human and parasites. The grade of connectivity for each node in the networks and functional annotations have dissimilarity between species. These findings denoted extensive species-specific network renewing that can likely be associated to specific protein evolution in each system.

To date, PPI networks related to mRNA polyadenylation have been poorly described in protozoan parasites. The architecture of predicted PPI networks confirms the link between the polyadenylation process and other events related to gene expression regulation in both human and parasites. These relationships between proteins that participate in different steps of gene mRNA processing and export have been experimentally demonstrated. For example, distinct groups have showed the interaction of members of the human CFIm complex with the polyadenylation factors PAP, PABP, and FIP1 [67–69], hClp1, U2AF, and snRNP U1 involved in splicing [63,70,71,72], the cap binding protein CBP20 [55], as well as Thoc5, Mex67-Mtr2, and Tap-p15 that participate in mRNA nuclear export [73]. In *E. histolytica*, we reported the interaction between CFIm25 and PAP [8]. Considering that grouping proteins with functional annotations contribute to a better identification of central functions and therefore lethal proteins [74], our results clearly evidenced that proteins involved in different steps of gene expression are prospective parasite targets.

Interestingly, the significant differences in PPI networks corresponding to parasites and the human host can be exploited to specifically target a pathogen protein. Network ‘hubs’ are highly connected proteins with many PPIs (high node degree); they are therefore likely to exert a higher influence on network function via multiple interactions [51]. The polyadenylation factor CPSF160 presents the highest degree of connectivity in the human PPI network, while central hubs are represented by PAP, WDR33, CPSF30 in *E. histolytica, T. vaginalis*, and *T. cruzi*, respectively. Additionally, central hubs do not correspond to polyadenylation factors in the other parasites. Computational predictions of PPI networks have allowed the identification of key proteins for cell survival [74] and potential drug targets against parasites [75]. Considering that proteins with a high degree of connectivity should be the most essential proteins [76,77], our results suggest that targeting PAP, WDR33, and CPSF30 could have an effect on parasite survival. Interestingly, some of these proteins have already been described as biochemical target. Notably, Hericks et al. reported that CPSF30 depletion affect polyadenylation and survival in *T. brucei* [13]. Moreover, CFIm25, CPSF30 (CPSF4), and CPSF73 (CPSF3) have been identified as potential therapeutic targets in *E. histolytica, T. brucei, T. gondii*, and *P. falciparum* [11–16]. Further experimental assays are required to validate the biological importance of central hubs proteins predicted here. Finally, it has also been described that interacting proteins have a correlated expression [51]. Congruently, we demonstrated the modulated expression of parasite polyadenylation proteins in response to diverse growth conditions, which suggests their functional interaction in agreement with PPI networks and strengthens the impact of targeting polyadenylation factors on parasite biology and survival.

Network ‘bottlenecks’ are proteins with high betweenness centrality; they regulate the flow of signaling information and therefore, represent central points for communication in an interaction network [51]. In the human PPI network, the connection between the bottleneck proteins PP1CC and BRCA1, agrees with a previous report showing that the tumor suppressor BRCA1 protein interacts with the polyadenylation protein CstF50 to promote deadenylation activity of the poly(A)-specific ribonuclease PARN during DNA damage, leading to RNA degradation [78]. In *E. histolytica* taken as a working model, the identification of different bottleneck proteins and connections between both PPI modules is a relevant finding that could help to detect parasite targets. Indeed, the silencing of the bottleneck protein CFIm25 largely affected gene expression in genes of both PPI modules, including Ehnopp34, the bottleneck protein of the second module. Additionally, changes in the expression of the central hub protein PAP and interacting polyadenylation factors EhCFIm25, EhCPSF1, EhCLP1, and EhRBPP6, confirms the relevance of polyadenylation genes for parasite survival, growth, and stress adaptation. Congruently with the relevance of these proteins with high betweenness centrality in the PPI network, we previously demonstrated that EhCFIm25 silencing inhibits parasite survival and virulence. Notably, we hypothesized that the greater effect of EhCFIm25 capture by specific RNA aptamers versus* EhCFIm25* gene silencing on *E. histolytica* proliferation and death was related to the concomitant capture of EhCFIm25 interacting proteins [11,12]. With the present results, we corroborate that the removal or capture of the bottleneck node CFIm25 broke down communication links between both modules of the PPI networks, affecting the expression of relevant connected proteins, which resulted to be fatal for the pathogen.

By correlating the consequences of the absence of individual yeast proteins with the number of their PPI, it has been shown that highly connected proteins with a central role in the network organization and topology, i.e. hubs and bottleneck proteins, are potentially three times more essential than proteins with a reduced number of PPI [76]. Using an integrative network approach, Alam et al. recently identified 86 hub proteins in tuberculosis and noncommunicable diseases and demonstrated the relation between drugs and these apparently unrelated targets and pathways, supporting the assumption that the most highly connected proteins in the cell are the most important for its survival [79]. The positive correlation between lethality and connectivity in PPI network therefore represents an interesting strategy to identify pathogen proteins that could represent new therapeutic targets for parasite controls. The identification of EhPAP as a hub protein suggests that its removal would strongly affect *E. histolytica* survival, as we observed for EhCFIm25. Further experimental assays are required to confirm the relevance of EhPAP as a new therapeutic target.

The reliability of the *E. histolytica* PPI was assessed by two complementary approaches: a molecular docking study of the EhCFIm25–Ehnopp34 interaction and a CLIP assay followed coupled to a proteomic analysis. The 3D models of EhCFIm25 and Ehnopp34 have been recently published in the AlphaFold platform [80,81]. Interestingly, the Nudix and RRM domains of EhCFIm25 and Ehnopp34 fold in a very similar way in our relaxed EhCFIm25 and Ehnopp34 models with RMSD values of 0.485 and 0.797 Å, respectively. In contrast, the most flexible regions, namely the N- and C-terminal of EhCFIm25 and the C-terminal of Ehnopp34 show significant differences with RMSD values up to 2.545 Å. However, their prediction is highly uncertain according to the trust report of AlphaFold (<60% in average for these regions). Therefore, we consider that the optimization of EhCFIm5 and Ehnopp43 structures by molecular dynamics simulations gave us better models for docking study. The connection between EhCFIm25 and Ehnopp34 was supported by two complementary computational analysis, the PPI network prediction, and the docking simulation. However, it is necessary to perform specific experiments to confirm this interaction. Nopp proteins are nucleolar phosphoproteins that shuttle between nucleus and cytoplasm. Nopp34, also known as NIFK or MKI67IP, interacts with the forkhead-associated domain of human Ki67. It presents an RNA recognition motif (RRM) at the N-terminus and a FHA Ki67 binding domain at the C-terminus, suggesting that it could be participating in mitotic chromosome organization [82]. The Ehnopp34 sequence contains only half the residues compared with that of the Hsnopp34, which has 293 residues; thus, it lacks the C-terminus, which has been shown to bind to FHA domains [52,82]. A sequence comparison between Ehnopp34 and Hsnopp34 showed 52.3% similarity between the RRM domains, and 32.2% identity (data not shown). The lack of the C-terminal domain in Ehnopp34 suggests that it might participate in different functions than Hsnopp34, in which the RRM domain is relevant. This domain has been found in proteins with diverse functions, such as pre-mRNA polyadenylation, for example, CstF-64 [83], splicing [84], mRNA stability [85], RNA editing [86], among other functions. Interestingly, it has been proposed that RRM domain-containing proteins could also participate in the recruitment of other proteins for the formation of RNA processing complexes. As discussed, larger CFIm components have not been identified in the amoeba machinery compared with the heterotetrameric complex described for humans, suggesting that other proteins could be supplying the function of the missing subunits. Study of the human CFIm25-CFIm68 complex showed that RNA association to the CFIm25 subunit is enhanced by the presence of the CFIm68 subunit, possibly facilitating RNA looping through the RRM domain so that the nucleotide sequences that are recognized by the CFIm25 subunit can be positioned in the binding pocket [55]. The role of RRM domains in this process appears to be fundamental, suggesting that proteins with RRM domains that have the ability to associate with the CFIm25 subunit could favor RNA binding to the binding pocket present in the Nudix domain of CFIm25. Our molecular docking results suggest that Ehnopp34 could bind to EhCFIm25 in a similar way to that reported for the human CFIm68 subunit, suggesting that this protein could facilitate the association of RNA to the EhCFIm25 monomers of the complex in *E. histolytica*.

The proteomic analysis of nuclear proteins interacting with EhCFIm25 led to the identification of the proteins related to the same pathways previously described in the *E. histolytica* PPI network. Particularly, IP and bioinformatics approaches identified proteins related to (1) ribosomes biogenesis (IP: 60S ribosomal protein L1, ribosomal protein S24; PPI network: 60S ribosomal protein, ribosome assembly factor mrt4, ribosome production factor 2 homolog, ribosome biogenesis protein NSA2 homolog); (2) WD domain contaning proteins; and (3) Transcription (IP: NEDD4-binding protein 2, elongation factor 1-α; PPI:tTranscription initiation factor SPT5, RNA-binding protein, transcription initiation protein SPT4). Therefore, the proteomic analysis validates our computational approach and confirms the relationship among the different events of gene expression regulation in *E. histolytica*. Additionally, the interaction of EhCFIm25 with Cys-peroxiredoxin and 60S ribosomal protein agrees with a previous report in *P. falciparum* that evidenced interactions between Cys-peroxiredoxins and proteins involved in translation [87]. Both antibodies used in the CLIP assays are polyclonal antibodies; therefore, it is possible that they bind to several and distinct epitopes in EhCFIm25, affecting its interaction with other proteins, which may explain the immunoprecipitation of different EhCFIm25-interacting proteins in both experiments; this is also an opportunity to expand the interactome.

The fact that EhCFIm25 was not detected in the CLIP assays is likely to be related to the protein identification protocol. Mass spectrometry works by measuring the relative intensity of the proteins; in such analyses, intensity peak ratios are treated as abundance ratios of the respective molecule. However, the technique has its limitations when studying protein complexes. Since the signal intensities of analytes of similar size can significantly distort the ratio of peaks due to the unequal signal response of the different analytes, this can make it difficult to analyze when trying to identify large molecular complex [88]. Therefore, because of the number of proteins bound to the complex, the EhCFIm25 signal was distorted, and the MS immediately discarded its identification. The absence of EhCFIm25 and other expected nuclear proteins, such as EhPAP that has been demonstrated to interact with EhCFIn25 [8] can also be related to the low abundance of true nuclear protein and their under-representation in proteomic studies by high-throughput techniques, since as we said above, the most abundant proteins determine the limit of detection for the less abundant ones. Thus, we cannot consider data of our CLIP assays as a complete illustration of EhCFIm25 interactome. It is possible that other experimental strategies, such as yeast two-hybrids system, aptoprecipitation with anti-EhCFIm25 aptamers, or *in vivo* UV-CLIP in EhCFIm25 overexpressing trophozoites, would provide a better understanding of the nuclear proteins interacting with EhCFIm25 in *E. histolytica*.

Surprisingly, the proteomic analysis of EhCFIm25 interactome also identified proteins that are typically considered as cytoplasmic. A similar observation has been previously reported for other nuclear proteomes [89,90]. The high abundance of these supposedly cytoplasmic proteins in the nuclear fraction suggest they might have moonlight functions. Congruently, several of the proteins related to energy metabolism, cell redox homeostasis and response to protein folding stress, have been described as moonlight proteins with multiple functions in distinct cellular compartments in *E. histolytica*. Thus, thioredoxin regulates the activity of the UDP-glucose pyrophosphorylase through post-translational modification [91]. Thioredoxin was detected in both the cytoplasm and the nucleus of trophozoites, where it plays distinct roles in a two-step mechanism of redox regulation of transcription factor NF-κB [92]. Peroxiredoxin was localized in the nucleus and cytoplasm of *E. moshkovskii*, trophozoites [93], while it was found in the nucleus and the membrane in *E. histolytica* [94]. GAPDH participates in telomere maintenance in the nucleus of human lung carcinoma cells [95]; under stresses, the translocation of glyceraldehyde-3-phosphate dehydrogenase from cytoplasm to the nucleus is regulated by acetylation [96]. In *E. histolytica*, GAPDH is modified by ADP-ribosylation and secreted to the extracellular medium where it may play an important role in ameba survival or in interaction with host cells or molecules [97]. This first attempt to identify EhCFIm25 interactome confirms the existence of close relationships among proteins involved in gene expression regulation and evidenced new links between gene expression and metabolism though connections with moonlight proteins in *E. histolytica*. Interestingly, enzymes that participate in biochemical pathways generating energy, reducing power and biosynthetic intermediates necessary for cell survival, have also been involved in ‘moonlighting’ as RNA-binding proteins in mammalians cells. Some authors have proposed the REM (RNA-enzyme and metabolite) network hypothesis in which RNA is considered as a key molecule that assembles all the enzymes of a particular metabolic pathway to promote the metabolic activity. It has also been suggested that these moonlight proteins could connect intermediary metabolism with RNA biology and posttranscriptional gene regulation [98,99]. In this work, parasite cultures were UV cross-linked prior to obtain nuclear proteins and perform immunoprecipitation assays with anti-EhCFIm25 antibodies, to ensure precipitation of RNA-binding proteins, including EhCFIm25 and proteins interacting to EhCFIm25. Immunoprecipitates also contain RNA molecules, then proteins identified by LC-ESI-HDMSE analyses can include other RNA-binding proteins in close proximity with EhCFIm25. We hypothesize that some of these proteins may correspond to moonlight proteins described above. However, additional experiments are required to confirm these assumptions in *E. histolytica*.

## Conclusion

Taken altogether, our results established the high potential of computational approaches based on comparative genomics and interactomics to predict PPI networks and identify key proteins for network function, which may open a new horizon for the characterization of new therapeutic targets in protozoan parasites that affect human health, such as EhCFIm25 in *E. histolytica*. Additionally, they confirmed the existence of important relationships between mRNA polyadenylation and other molecular events involved in gene expression regulation in *E. histolytica*. Finally, the possible role of RNA as a scaffolding molecule that connects RNA biology and posttranscriptional gene regulation with intermediary metabolism requires a specific attention to better understand *E. histolytica* biology.

## Supplementary Material

Supplementary Figures S1-S3

Supplementary Table S1

## Data Availability

The mass spectrometry proteomic data have been deposited to the ProteomeXchange Consortium via the PRIDE [50] partner repository with the dataset identifier PXD033620.

## Abbreviations

CPSF: cleavage and polyadenylation specificity factor
FDR: false discovery rate
MD: molecular dynamics
PME: Particle Mesh Ewald
PPI: protein–protein interactions
REM: RNA-enzyme and metabolite

## References

[1] Jalkanen A.L., Coleman S.J. and Wilusz J. (2014) Determinants and implications of mRNA poly(A) tail size–does this protein make my tail look big? Sem. Cell Dev. Biol. 34, 24–32 10.1016/j.semcdb.2014.05.018 PMC4163081 24910447

[2] Xiang K., Tong L. and Manley J.L. (2014) Delineating the structural blueprint of the pre-mRNA 3′-end processing machinery. Mol. Cell. Biol. 34, 1894–1910 10.1128/MCB.00084-14 24591651 PMC4019069

[3] Neve J., Patel R., Wang Z., Louey A. and Furger A.M. (2017) Cleavage and polyadenylation: ending the message expands gene regulation. RNA Biol. 14, 865–890 10.1080/15476286.2017.1306171 28453393 PMC5546720

[4] Rambout X. and Maquat L.E. (2020) The nuclear cap-binding complex as choreographer of gene transcription and pre-mRNA processing. Genes Dev. 34, 1113–1127 10.1101/gad.339986.120 32873578 PMC7462061

[5] Nourse J., Spada S. and Danckwardt S. (2020) Emerging roles of RNA 3′-end cleavage and polyadenylation in pathogenesis, diagnosis and therapy of human disorders. Biomolecules 10, 915 10.3390/biom10060915 32560344 PMC7356254

[6] Ospina-Villa J.D., Tovar-Ayona B.J., López-Camarillo C., Soto-Sánchez J., Ramírez-Moreno E., Castañón-Sánchez C.A. et al. (2020) mRNA polyadenylation machineries in intestinal protozoan parasites. J. Eukaryot. Microbiol. 67, 306–320 10.1111/jeu.12781 31898347

[7] García-Vivas J., López-Camarillo C., Azuara-Liceaga E., Orozco E. and Marchat L.A. (2005) Entamoeba histolytica: cloning and expression of the poly(A) polymerase EhPAP. Exp. Parasitol. 110, 226–232 10.1016/j.exppara.2005.02.017 15955317

[8] Pezet-Valdez M., Fernández-Retana J., Ospina-Villa J.D., Ramírez-Moreno M.E., Orozco E., Charcas-López S. et al. (2013) The 25 kDa subunit of cleavage factor Im Is a RNA-binding protein that interacts with the poly(A) polymerase in Entamoeba histolytica. PloS ONE 8, e67977 10.1371/journal.pone.0067977 23840799 PMC3695940

[9] Ospina-Villa J.D., Zamorano-Carrillo A., Lopez-Camarillo C., Castañon-Sanchez C.A., Soto-Sanchez J., Ramirez-Moreno E. et al. (2015) Amino acid residues Leu135 and Tyr236 are required for RNA binding activity of CFIm25 in Entamoeba histolytica. Biochimie 115, 44–51 10.1016/j.biochi.2015.04.017 25941172

[10] Salgado-Martínez A.I., Avila-Bonilla R.G., Ramírez-Moreno E., Castañón-Sánchez C.A., López-Camarillo C. and Marchat L.A. (2021) Unraveling the relevance of the polyadenylation factor EhCFIm25 in Entamoeba histolytica through proteomic analysis. FEBS Open. Bio. 11, 2819–2835 10.1002/2211-5463.13287 34486252 PMC8487052

[11] Ospina-Villa J.D., Guillen N., Lopez-Camarillo C., Soto-Sanchez J., Ramirez-Moreno E., Garcia-Vazquez R. et al. (2017) Silencing the cleavage factor CFIm25 as a new strategy to control Entamoeba histolytica parasite. J. Microbiol. 55, 783–791 10.1007/s12275-017-7259-9 28956353

[12] Ospina-Villa J.D., Dufour A., Weber C., Ramirez-Moreno E., Zamorano-Carrillo A., Guillen N. et al. (2018) Targeting the polyadenylation factor EhCFIm25 with RNA aptamers controls survival in Entamoeba histolytica. Sci. Rep. 8, 5720 10.1038/s41598-018-23997-w 29632392 PMC5890266

[13] Hendriks E.F., Abdul-Razak A. and Matthews K.R. (2003) tbCPSF30 depletion by RNA interference disrupts polycistronic RNA processing in Trypanosoma brucei. J. Biol. Chem. 278, 26870–26878 10.1074/jbc.M302405200 12746436

[14] Sidik S.M., Huet D., Ganesan S.M., Huynh M.H., Wang T., Nasamu A.S. et al. (2016) A Genome-wide CRISPR Screen in Toxoplasma Identifies Essential Apicomplexan Genes. Cell 166, 1423.e12–1435.e12 10.1016/j.cell.2016.08.019 27594426 PMC5017925

[15] Palencia A., Bougdour A., Brenier-Pinchart M.P., Touquet B., Bertini R.L., Sensi C. et al. (2017) Targeting Toxoplasma gondii CPSF3 as a new approach to control toxoplasmosis. EMBO Mol. Med. 9, 385–394 10.15252/emmm.201607370 28148555 PMC5331205

[16] Sonoiki E., Ng C.L., Lee M.C., Guo D., Zhang Y.K., Zhou Y. et al. (2017) A potent antimalarial benzoxaborole targets a Plasmodium falciparum cleavage and polyadenylation specificity factor homologue. Nat. Commun. 8, 14574 10.1038/ncomms14574 28262680 PMC5343452

[17] Begolo D., Vincent I.M., Giordani F., PoÈhner I., Witty M.J., Rowan T.G. et al. (2018) The trypanocidal benzoxaborole AN7973 inhibits trypanosome mRNA processing. PLoS Pathog. 14, e1007315 10.1371/journal.ppat.1007315 30252911 PMC6173450

[18] Murakami Y., Tripathi L.P., Prathipati P. and Mizuguchi K. (2017) Network analysis and in silico prediction of protein-protein interactions with applications in drug discovery. Curr. Opin. Struct. Biol. 44, 134–142 10.1016/j.sbi.2017.02.005 28364585

[19] LaCount D.J., Vignali M., Chettier R., Phansalkar A., Bell R., Hesselberth J.R. et al. (2005) A protein interaction network of the malaria parasite Plasmodium falciparum. Nature 438, 103–107 10.1038/nature04104 16267556

[20] Ramaprasad A., Pain A. and Ravasi T. (2012) Defining the protein interaction network of human malaria parasite Plasmodium falciparum. Genomics 99, 69–75 10.1016/j.ygeno.2011.11.006 22178265

[21] Gordon D.E., Jang G.M., Bouhaddou M., Xu J., Obernier K., White K.M. et al. (2020) A SARS-CoV-2 protein interaction map reveals targets for drug repurposing. Nature 583, 459–468 10.1038/s41586-020-2286-9 32353859 PMC7431030

[22] Dyer M.D., Murali T.M. and Sobral B.W. (2007) Computational prediction of host-pathogen protein-protein interactions. Bioinformatics 23, i159–i166 10.1093/bioinformatics/btm208 17646292

[23] Saha S., Sengupta K., Chatterjee P., Basu S. and Nasipuri M. (2018) Analysis of protein targets in pathogen-host interaction in infectious diseases: a case study on Plasmodium falciparum and Homo sapiens interaction network. Brief. Funct. Genomics 17, 441–450 29028886 10.1093/bfgp/elx024

[24] Acharya D. and Dutta T.K. (2021) Elucidating the network features and evolutionary attributes of intra- and interspecific protein-protein interactions between human and pathogenic bacteria. Sci. Rep. 11, 190 10.1038/s41598-020-80549-x 33420198 PMC7794237

[25] Gouy M., Guindon S. and Gascuel O. (2010) SeaView version 4: A multiplatform graphical user interface for sequence alignment and phylogenetic tree building. Mol. Biol. Evol. 27, 221–224 10.1093/molbev/msp259 19854763

[26] Lefort V., Longueville J.E. and Gascuel O. (2017) SMS: Smart Model Selection in PhyML. Mol. Biol. Evol. 34, 2422–2424 10.1093/molbev/msx149 28472384 PMC5850602

[27] Naiyer S., Kaur D., Ahamad J., Singh S.S., Singh Y.P., Thakur V. et al. (2019) Transcriptomic analysis reveals novel downstream regulatory motifs and highly transcribed virulence factor genes of Entamoeba histolytica. BMC Genomics 20, 206 10.1186/s12864-019-5570-z 30866809 PMC6416950

[28] Hon C.C., Weber C., Sismeiro O., Proux C., Koutero M., Deloger M. et al. (2013) Quantification of stochastic noise of splicing and polyadenylation in Entamoeba histolytica. Nucleic Acids Res. 41, 1936–1952 10.1093/nar/gks1271 23258700 PMC3561952

[29] Roth A., Adapa S.R., Zhang M., Liao X., Saxena V., Goffe R. et al. (2018) Unraveling the Plasmodium vivax sporozoite transcriptional journey from mosquito vector to human host. Sci. Rep. 8, 12183 10.1038/s41598-018-30713-1 30111801 PMC6093925

[30] Gural N., Mancio-Silva L., Miller A.B., Galstian A., Butty V.L., Levine S.S. et al. (2018) In vitro culture, drug sensitivity, and transcriptome of plasmodium vivax hypnozoites. Cell Host Microbe. 23, 395.e4–406.e4 10.1016/j.chom.2018.01.002 29478773 PMC8048090

[31] Vivax Sporozoite Consortium (2019) Transcriptome and histone epigenome of Plasmodium vivax salivary-gland sporozoites point to tight regulatory control and mechanisms for liver-stage differentiation in relapsing malaria. Int. J. Parasitol. 49, 501–513 10.1016/j.ijpara.2019.02.007 31071319 PMC9973533

[32] Siegel T.N., Hekstra D.R., Wang X., Dewell S. and Cross G.A. (2010) Genome-wide analysis of mRNA abundance in two life-cycle stages of Trypanosoma brucei and identification of splicing and polyadenylation sites. Nucleic Acids Res. 38, 4946–4957 10.1093/nar/gkq237 20385579 PMC2926603

[33] Xu J. and Wang S. (2019) Analysis of distance-based protein structure prediction by deep learning in CASP13. Proteins 87, 1069–1081 10.1002/prot.25810 31471916

[34] Waterhouse A., Bertoni M., Bienert S., Studer G., Tauriello G., Gumienny R. et al. (2018) SWISS-MODEL: homology modelling of protein structures and complexes. Nucleic Acids Res. 46, W296–W303 10.1093/nar/gky427 29788355 PMC6030848

[35] Shuid A.N., Kempster R. and McGuffin L.J. (2017) ReFOLD: a server for the refinement of 3D protein models guided by accurate quality estimates. Nucleic Acids Res. 45, W422–W428 10.1093/nar/gkx249 28402475 PMC5570150

[36] Bowie J.U., Lüthy R. and Eisenberg D. (1991) A method to identify protein sequences that fold into a known three-dimensional structure. Science 253, 164–170 10.1126/science.1853201 1853201

[37] Laskowski R.A., MacArthur M.W., Moss D.S. and Thornton J.M. (1993) PROCHECK: a program to check the stereochemical quality of protein structures. J. Appl. Cryst. 26, 283–291 10.1107/S0021889892009944

[38] Abraham M.J., Murtola T., Schulz R., Paĺl S., Smith J.C., Hess B. et al. (2015) GROMACS: High performance molecular simulations through multi-level parallelism from laptops to supercomputers. SoftwareX 1-2, 19–25 10.1016/j.softx.2015.06.001

[39] Jorgensen W.L., Maxwell D.S. and Tirado-Rives J. (1996) Development and testing of the OPLS all-atom force field on conformational energetics and properties of organic liquids. J. Am. Chem. Soc. 118, 11225–11236 10.1021/ja9621760

[40] Christoffer C., Chen S., Bharadwaj V., Aderinwale T., Kumar V., Hormati M. et al. (2021) LZerD webserver for pairwise and multiple protein-protein docking. Nucleic Acids Res. 49, W359–W365 10.1093/nar/gkab336 33963854 PMC8262708

[41] Diamond L.S., Harlow D.R. and Cunnick C.C. (1978) A new medium for the axenic cultivation of Entamoeba histolytica and other Entamoeba. Trans. R. Soc. Trop. Med. Hyg. 72, 431–432 10.1016/0035-9203(78)90144-X 212851

[42] Narayanasamy R.K., Castañón-Sanchez C.A., Luna-Arias J.P., García-Rivera G., Avendaño-Borromeo B., Labra-Barrios M.L. et al. (2018) The Entamoeba histolytica TBP and TRF1 transcription factors are GAAC-box binding proteins, which display differential gene expression under different stress stimuli and during the interaction with mammalian cells. Parasit. Vectors 11, 153 10.1186/s13071-018-2698-7 29514716 PMC5842622

[43] Olivos-García A., Nequiz M., Liceaga S., Mendoza E., Zúñiga P., Cortes A. et al. (2018) Complement is a rat natural resistance factor to amoebic liver infection. Biosci. Rep. 38, BSR20180713 10.1042/BSR20180713 30201693 PMC6167500

[44] Penuliar G.M., Furukawa A., Nakada-Tsukui K., Husain A., Sato D. and Nozaki T. (2012) Transcriptional and functional analysis of trifluoromethionine resistance in Entamoeba histolytica. J. Antimicrob. Chemother. 67, 375–386 10.1093/jac/dkr484 22110087

[45] Valdés J., Nozaki T., Sato E., Chiba Y., Nakada-Tsukui K., Villegas-Sepúlveda N. et al. (2014) Proteomic analysis of Entamoeba histolytica in vivo assembled pre-mRNA splicing complexes. J. Proteomics 111, 30–45 10.1016/j.jprot.2014.07.027 25109466

[46] de la Cruz O.H., Muñiz-Lino M., Guillén N., Weber C., Marchat L.A., López-Rosas I. et al. (2014) Proteomic profiling reveals that EhPC4 transcription factor induces cell migration through up-regulation of the 16-kDa actin-binding protein EhABP16 in Entamoeba histolytica. J. Proteomics 111, 46–58 10.1016/j.jprot.2014.03.041 24721673

[47] López-Rosas I., Marcha L.A., Olvera B.G., Guillen N., Weber C., Hernández de la Cruz O. et al. (2014) Proteomic analysis identifies endoribouclease EhL-PSP and EhRRP41 exosome protein as novel interactors of EhCAF1 deadenylase. J. Proteomics 111, 59–73 10.1016/j.jprot.2014.06.019 24998979

[48] Ramírez-Flores C.J., Cruz-Mirón R., Mondragón-Castelán M.E., González-Pozos S., Ríos-Castro E. and Mondragón-Flores R. (2019) Proteomic and structural characterization of self-assembled vesicles from excretion/secretion products of Toxoplasma gondii. J. Proteomics 208, 103490 10.1016/j.jprot.2019.103490 31434009

[49] Félix-Contreras C., Alba-Fierro C.A., Ríos-Castro E., Luna-Martínez F., Cuéllar-Cruz M. and Ruiz-Baca E. (2020) Proteomic analysis of Sporothrix schenckii cell wall reveals proteins involved in oxidative stress response induced by menadione. Microbial. Pathog. 141, 103987 10.1016/j.micpath.2020.103987 31962184

[50] Perez-Riverol Y., Csordas A., Bai J., Bernal-Llinares M., Hewapathirana S., Kundu D.J. et al. (2019) The PRIDE database and related tools and resources in 2019: improving support for quantification data. Nucleic Acids Res. 47:, D442–D450 10.1093/nar/gky1106 30395289 PMC6323896

[51] Yu H., Kim P.M., Sprecher E., Trifonov V. and Gerstein M. (2007) The importance of bottlenecks in protein networks: correlation with gene essentiality and expression dynamics. PLoS Comput. Biol. 3, e59 10.1371/journal.pcbi.0030059 17447836 PMC1853125

[52] Emsley P. and Cowtan K. (2004) Coot: model-building tools for molecular graphics. Acta Crystallogr. D. Biol. Crystallogr. 60, 2126–2132 10.1107/S0907444904019158 15572765

[53] Li H., Byeon I.J., Ju Y. and Tsai M.D. (2004) Structure of human Ki67 FHA domain and its binding to a phosphoprotein fragment from hNIFK reveal unique recognition sites and new views to the structural basis of FHA domain functions. J. Mol. Biol. 335, 371–381 10.1016/j.jmb.2003.10.032 14659764

[54] Yang Q., Gilmartin G.M. and Doublié S. (2010) Structural basis of UGUA recognition by the Nudix protein CFI(m)25 and implications for a regulatory role in mRNA 3′ processing. Proc. Natl. Acad. Sci. U.S.A. 107, 10062–10067 10.1073/pnas.1000848107 20479262 PMC2890493

[55] Yang Q., Coseno M., Gilmartin G.M. and Doublié S. (2011) Crystal structure of a human cleavage factor CFI(m)25/CFI(m)68/RNA complex provides an insight into poly(A) site recognition and RNA looping. Structure 19, 368–377 10.1016/j.str.2010.12.021 21295486 PMC3056899

[56] Saavedra E., Encalada R., Vázquez C., Olivos-García A., Michels P. and Moreno-Sánchez R. (2019) Control and regulation of the pyrophosphate-dependent glucose metabolism in Entamoeba histolytica. Mol. Biochem. Parasitol. 229, 75–87 10.1016/j.molbiopara.2019.02.002 30772421

[57] Jeelani G. and Nozaki T. (2016) Entamoeba thiol-based redox metabolism: A potential target for drug development. Mol. Biochem. Parasitol. 206, 39–45 10.1016/j.molbiopara.2016.01.004 26775086

[58] Jäger A.V., De Gaudenzi J.G., Cassola A., D'Orso I. and Frasch A.C. (2007) mRNA maturation by two-step trans-splicing/polyadenylation processing in trypanosomes. Proc. Natl. Acad. Sci. U.S.A. 104, 2035–2042 10.1073/pnas.0611125104 17267594 PMC1892994

[59] Fuentes V., Barrera G., Sánchez J., Hernández R. and López-Villaseñor I. (2012) Functional analysis of sequence motifs involved in the polyadenylation of Trichomonas vaginalis mRNAs. Eukaryot. Cell 11, 725–734 10.1128/EC.05322-11 22467744 PMC3370463

[60] Stevens A.T., Howe D.K. and Hunt A.G. (2018) Characterization of mRNA polyadenylation in the apicomplexa. PloS ONE 13, e0203317 10.1371/journal.pone.0203317 30161237 PMC6117058

[61] Schönemann L., Kühn U., Martin G., Schäfer P., Gruber A.R., Keller W. et al. (2014) Reconstitution of CPSF active in polyadenylation: recognition of the polyadenylation signal by WDR33. Genes Dev. 28, 2381–2393 10.1101/gad.250985.114 25301781 PMC4215183

[62] Kubo T., Wada T., Yamaguchi Y., Shimizu A. and Handa H. (2006) Knock-down of 25 kDa subunit of cleavage factor Im in Hela cells alters alternative polyadenylation within 3′-UTRs. Nucleic Acids Res. 34, 6264–6271 10.1093/nar/gkl794 17098938 PMC1669743

[63] Kim S., Yamamoto J., Chen Y., Aida M., Wada T., Handa H. et al. (2010) Evidence that cleavage factor Im is a heterotetrameric protein complex controlling alternative polyadenylation. Genes Cells 15, 1003–1013 10.1111/j.1365-2443.2010.01436.x 20695905

[64] Dominski Z., Yang X.C. and Marzluff W.F. (2005) The polyadenylation factor CPSF-73 is involved in histone-pre-mRNA processing. Cell 123, 37–48 10.1016/j.cell.2005.08.002 16213211

[65] Kühn U., Gündel M., Knoth A., Kerwitz Y., Rüdel S. and Wahle E. (2009) Poly(A) tail length is controlled by the nuclear poly(A)-binding protein regulating the interaction between poly(A) polymerase and the cleavage and polyadenylation specificity factor. J. Biol. Chem. 284, 22803–22814 10.1074/jbc.M109.018226 19509282 PMC2755688

[66] Darmon S.K. and Lutz C.S. (2012) mRNA 3′ end processing factors: a phylogenetic comparison. Comp. Funct. Genomics 2012, 876893 10.1155/2012/876893 22400011 PMC3287031

[67] Kim H. and Lee Y. (2001) Interaction of poly(A) polymerase with the 25-kDa subunit of cleavage factor I. Biochem. Biophys. Res. Commun. 289, 513–518 10.1006/bbrc.2001.5992 11716503

[68] Dettwiler S., Aringhieri C., Cardinale S., Keller W. and Barabino S.M.L. (2004a) Distinct sequence motifs within the 68-kDa subunit of cleavage factor Im mediate RNA binding, protein-protein interactions, and subcellular localization. J. Biol. Chem. 279, 35788–35797 10.1074/jbc.M403927200 15169763

[69] Venkataraman K., Brown K.M. and Gilmartin G.M. (2005) Analysis of a noncanonical poly(A) site reveals a tripartite mechanism for vertebrate poly(A) site recognition. Genes Dev. 19, 1315–1327 10.1101/gad.1298605 15937220 PMC1142555

[70] de Vries H. (2000) Human pre-mRNA cleavage factor IIm contains homologs of yeast proteins and bridges two other cleavage factors. EMBO J. 19, 5895–5904 10.1093/emboj/19.21.5895 11060040 PMC305781

[71] Awasthi S. and Alwine J.C. (2003) Association of polyadenylation cleavage factor I with U1 snRNP. RNA 9, 1400–1409 10.1261/rna.5104603 14561889 PMC1287061

[72] Weitzer S. and Martinez J. (2007) The human RNA kinase hClp1 is active on 3′ transfer RNA exons and short interfering RNAs. Nature 447:, 222–226 10.1038/nature05777 17495927

[73] Katahira J., Okuzaki D., Inoue H., Yoneda Y., Maehara K. and Ohkawa Y. (2013) Human TREX component Thoc5 affects alternative polyadenylation site choice by recruiting mammalian cleavage factor I. Nucleic Acids Res. 41, 7060–7072 10.1093/nar/gkt414 23685434 PMC3737531

[74] Tew K.L., Li X.L. and Tan S.H. (2007) Functional centrality: detecting lethality of proteins in protein interaction networks. Genome Inform. 19, 166–177 10.1142/9781860949852_0015 18546514

[75] Swann J., Jamshidi N., Lewis N.E. and Winzeler E.A. (2015) Systems analysis of host-parasite interactions. Wiley Interdiscip. Rev. Syst. Biol. Med. 7, 381–400 10.1002/wsbm.1311 26306749 PMC4679367

[76] Jeong H., Mason S.P., Barabási A.L. and Oltvai Z.N. (2001) Lethality and centrality in protein networks. Nature 411, 41–42 10.1038/35075138 11333967

[77] He X. and Zhang J. (2006) Why do hubs tend to be essential in protein networks? PLos Genet. 2, e88 10.1371/journal.pgen.0020088 16751849 PMC1473040

[78] Cevher M.A., Zhang X., Fernandez S., Kim S., Baquero J., Nilsson P. et al. (2010) Nuclear deadenylation/polyadenylation factors regulate 3′ processing in response to DNA damage. EMBO J. 29, 1674–1687 10.1038/emboj.2010.59 20379136 PMC2876964

[79] Alam A., Abubaker Bagabir H., Sultan A., Siddiqui M.F., Imam N., Alkhanani M.F. et al. (2022) An integrative network approach to identify common genes for the therapeutics in tuberculosis and its overlapping non-communicable diseases. Front. Pharmacol. 12, 770762 10.3389/fphar.2021.770762 35153741 PMC8829040

[80] Tunyasuvunakool K., Adler J., Wu Z., Green T., Zielinski M., Žídek A. et al. (2021) Highly accurate protein structure prediction for the human proteome. Nature 596, 590–596 10.1038/s41586-021-03828-1 34293799 PMC8387240

[81] Varadi M., Anyango S., Deshpande M., Nair S., Natassia C., Yordanova G. et al. (2022) AlphaFold Protein Structure Database: massively expanding the structural coverage of protein-sequence space with high-accuracy models. Nucleic Acids Res. 50, D439–D444 10.1093/nar/gkab1061 34791371 PMC8728224

[82] Takagi M., Sueishi M., Saiwaki T., Kametaka A. and Yoneda Y. (2001) A novel nucleolar protein, NIFK, interacts with the forkhead associated domain of Ki-67 antigen in mitosis. J. Biol. Chem. 276, 25386–25391 10.1074/jbc.M102227200 11342549

[83] Romeo V., Griesbach E. and Schümperli D. (2014) CstF64: cell cycle regulation and functional role in 3′ end processing of replication-dependent histone mRNAs. Mol. Cell. Biol. 34, 4272–4284 10.1128/MCB.00791-14 25266659 PMC4248742

[84] Kataoka N., Diem M.D., Yoshida M., Hatai C., Dobashi I., Dreyfuss G. et al. (2011) Specific Y14 domains mediate its nucleo-cytoplasmic shuttling and association with spliced mRNA. Sci. Rep. 1, 92 10.1038/srep00092 22355610 PMC3216578

[85] Kajjo S., Sharma S., Chen S., Brothers W.R., Cott M., Hasaj B. et al. (2022) PABP prevents the untimely decay of select mRNA populations in human cells. EMBO J. 41, e108650 10.15252/embj.2021108650 35156721 PMC8922270

[86] Galloway C.A., Kumar A., Krucinska J. and Smith H.C. (2010) APOBEC-1 complementation factor (ACF) forms RNA-dependent multimers. Biochem. Biophys. Res. Commun. 398, 38–43 10.1016/j.bbrc.2010.06.021 20541536 PMC2912146

[87] Brandstaedter C., Delahunty C., Schipper S., Rahlfs S., Yates J.R. 3rd and Becker K. (2019) The interactome of 2-Cys peroxiredoxins in Plasmodium falciparum. Sci. Rep. 9, 13542 10.1038/s41598-019-49841-3 31537845 PMC6753162

[88] Root K., Wittwer Y., Barylyuk K., Anders U. and Zenobi R. (2017) Insight into signal response of protein ions in native ESI-MS from the analysis of model mixtures of covalently linked protein oligomers. J. Am. Soc. Mass. Spectrom. 28, 1863–1875 10.1007/s13361-017-1690-3 28593376

[89] Goos C., Dejung M., Janzen C.J., Butter F. and Kramer S. (2017) The nuclear proteome of Trypanosoma brucei. PloS ONE 12, e0181884 10.1371/journal.pone.0181884 28727848 PMC5519215

[90] Briquet S., Ourimi A., Pionneau C., Bernardes J., Carbone A., Chardonnet S. et al. (2018) Identification of Plasmodium falciparum nuclear proteins by mass spectrometry and proposed protein annotation. PloS ONE 13, e0205596 10.1371/journal.pone.0205596 30379851 PMC6209197

[91] Martínez L.I., Piattoni C.V., Garay S.A., Rodrígues D.E., Guerrero S.A. and Iglesias A.A. (2011) Redox regulation of UDP-glucose pyrophosphorylase from Entamoeba histolytica. Biochimie 93, 260–268 10.1016/j.biochi.2010.09.019 20888387

[92] Hirota K., Murata M., Sachi Y., Nakamura H., Takeuchi J., Mori K. et al. (1999) Distinct roles of thioredoxin in the cytoplasm and in the nucleus. A two-step mechanism of redox regulation of transcription factor NF-kappaB. J. Biol. Chem. 274, 27891–27897 10.1074/jbc.274.39.27891 10488136

[93] Cheng X.J., Yoshihara E., Takeuchi T. and Tachibana H. (2004) Molecular characterization of peroxiredoxin from Entamoeba moshkovskii and a comparison with Entamoeba histolytica. Mol. Biochem. Parasitol. 138, 195–203 10.1016/j.molbiopara.2004.08.009 15555731

[94] Lyer L.R., Singh N., Verma A.K. and Paul J. (2014) Differential expression and immunolocalization of antioxidant enzymes in Entamoeba histolytica isolates during metronidazole stress. BioMed Res. Int. 2014, 704937 25013795 10.1155/2014/704937 PMC4074981

[95] Sundararaj K.P., Wood R.E., Ponnusamy S., Salas A.M., Szulc Z., Bielawska A. et al. (2004) Rapid shortening of telomere length in response to ceramide involves the inhibition of telomere binding activity of nuclear glyceraldehyde-3-phosphate dehydrogenase. J. Biol. Chem. 279, 6152–6162 10.1074/jbc.M310549200 14630908

[96] Ventura M., Mateo F., Serratosa J., Salaet I., Carujo S., Bachs O. et al. (2010) Nuclear translocation of glyceraldehyde-3-phosphate dehydrogenase is regulated by acetylation. Int. J. Biochem. Cell Biol. 42, 1672–1680 10.1016/j.biocel.2010.06.014 20601085

[97] Alvarez A.H., Martinez-Cadena G., Silva M.E., Saavedra E. and Avila E.E. (2007) Entamoeba histolytica: ADP-ribosylation of secreted glyceraldehyde-3-phosphate dehydrogenase. Exp. Parasitol. 117, 349–356 10.1016/j.exppara.2007.04.016 17586498

[98] Beckmann B.M., Castello A. and Medenbach J. (2016) The expanding universe of ribonucleoproteins: of novel RNA-binding proteins and unconventional interactions. Pflugers Archiv 468, 1029–1040 10.1007/s00424-016-1819-4 27165283 PMC4893068

[99] Ferreira L., Li A.M., Serafim T.L., Sobral M.C., Alpoim M.C. and Urbano A.M. (2020) Intermediary metabolism: An intricate network at the crossroads of cell fate and function. Biochim. Biophys. Acta Mol. Basis Dis. 1866, 165887 10.1016/j.bbadis.2020.165887 32599141

