# Supplementary material for: Comparative genomics and interactomics of polyadenylation factors for the prediction of new parasite targets: Entamoeba histolytica as a working model
Source: Biosci Rep. 2023 Feb 9;43(2):BSR20221911. doi: 10.1042/BSR20221911 (PMC9912109; doi:10.1042/BSR20221911)
Supplement: Supplementary Figures S1-S3 [file BSR-2022-1911_supp.pdf]

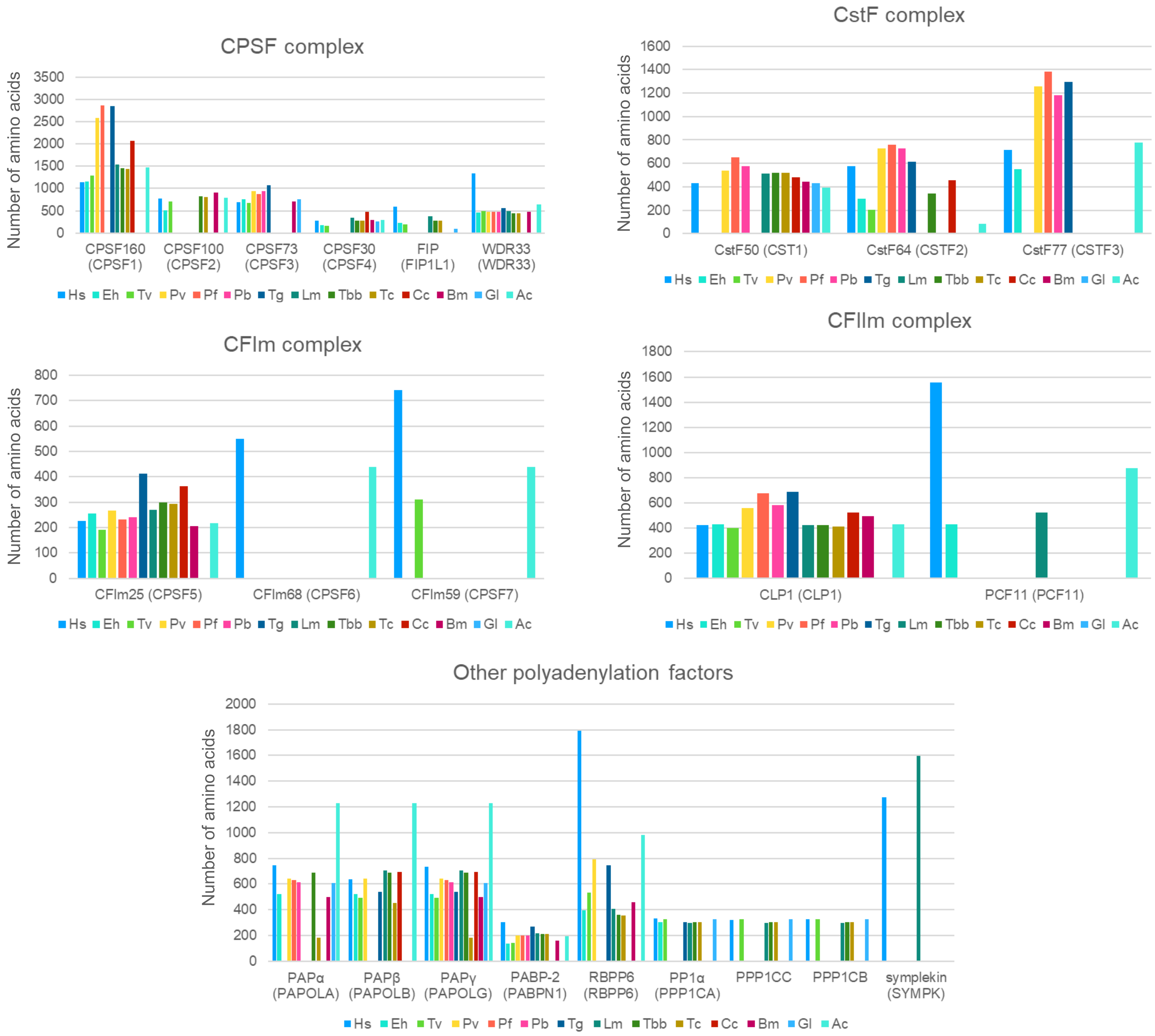

Supplementary Figure 1. Polyadenylation factors length (gene name is in parenthesis) in protozoan parasites and their human host. Each species corresponds to a specific color. H. sapiens (Hs), Entamoeba histolytica (Eh), Acanthamoeba castellani (Ac), Trichomonas vaginalis (Tv), Leishmania mexicana (Lm), Trypanosoma brucei brucei (Tbb), T. cruzi (Tc). Tozoplasma gondi (Tg), Plasmodium vivax (Pv), P. falciparum (Pf), Babesia microti (Bm), P. berghei (Pb), Cyclospora cayatenesis (Cc), and Giardia lamblia (Gl).

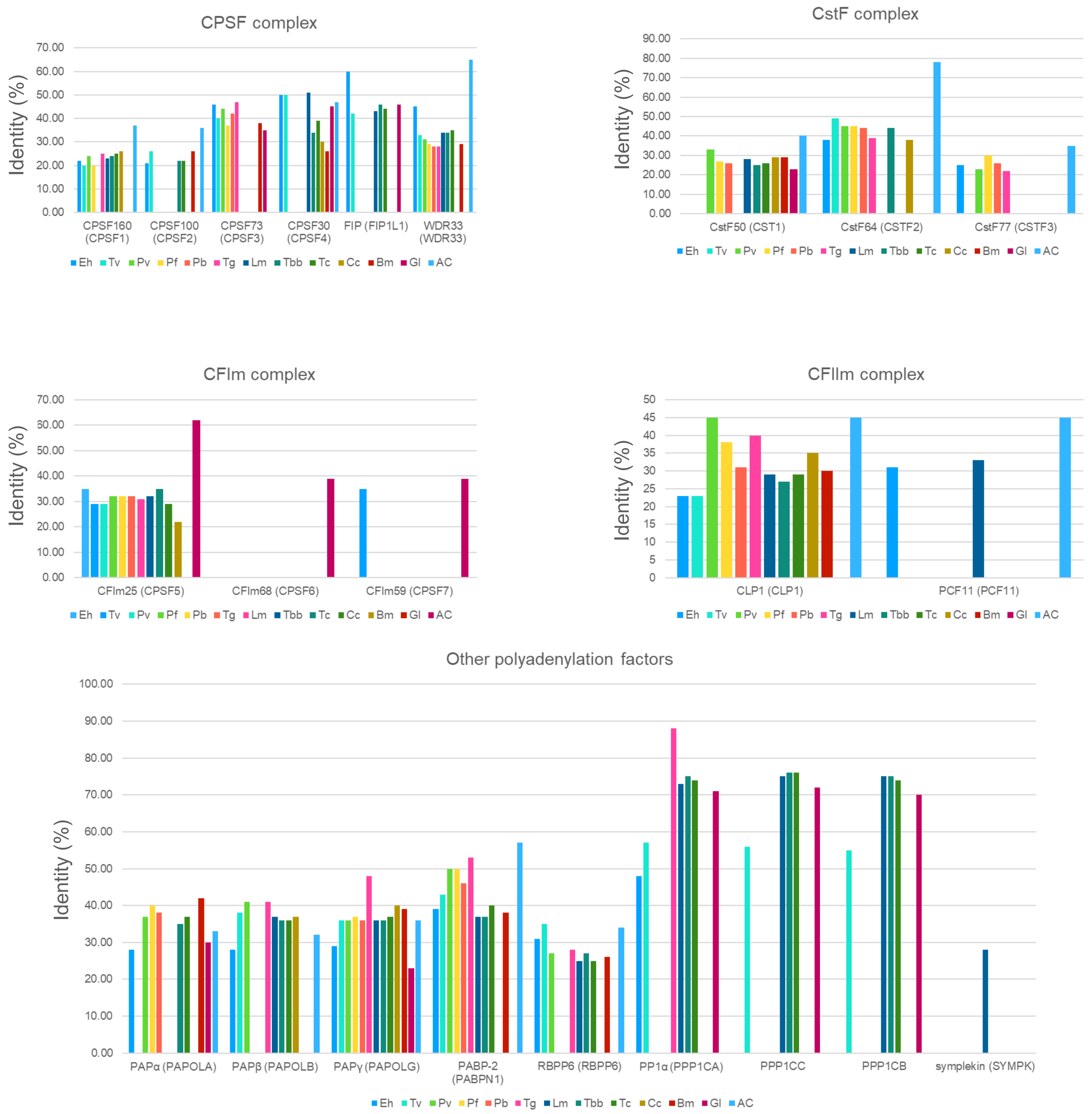

Supplementary Figure 2. Conservation of protein sequences in polyadenylation factors (gene name is in parenthesis). The protein sequence for each parasite factor was compared to the homologous human factor by BLAST analysis to obtain the percentage of identity. Each species corresponds to a specific color. H. sapiens (Hs), Entamoeba histolytica (Eh), Acanthamoeba castellani (Ac), Trichomonas vaginalis (Tv), Leishmania mexicana (Lm), Trypanosoma brucei brucei (Tbb), T. cruzi (Tc). Tozoplasma gondi (Tg), Plasmodium vivax (Pv), P. falciparum (Pf), Babesia microti (Bm), P. berghei (Pb), Cyclospora cayatenesis (Cc), and Giardia lamblia (Gl).

Supplementary figure 3

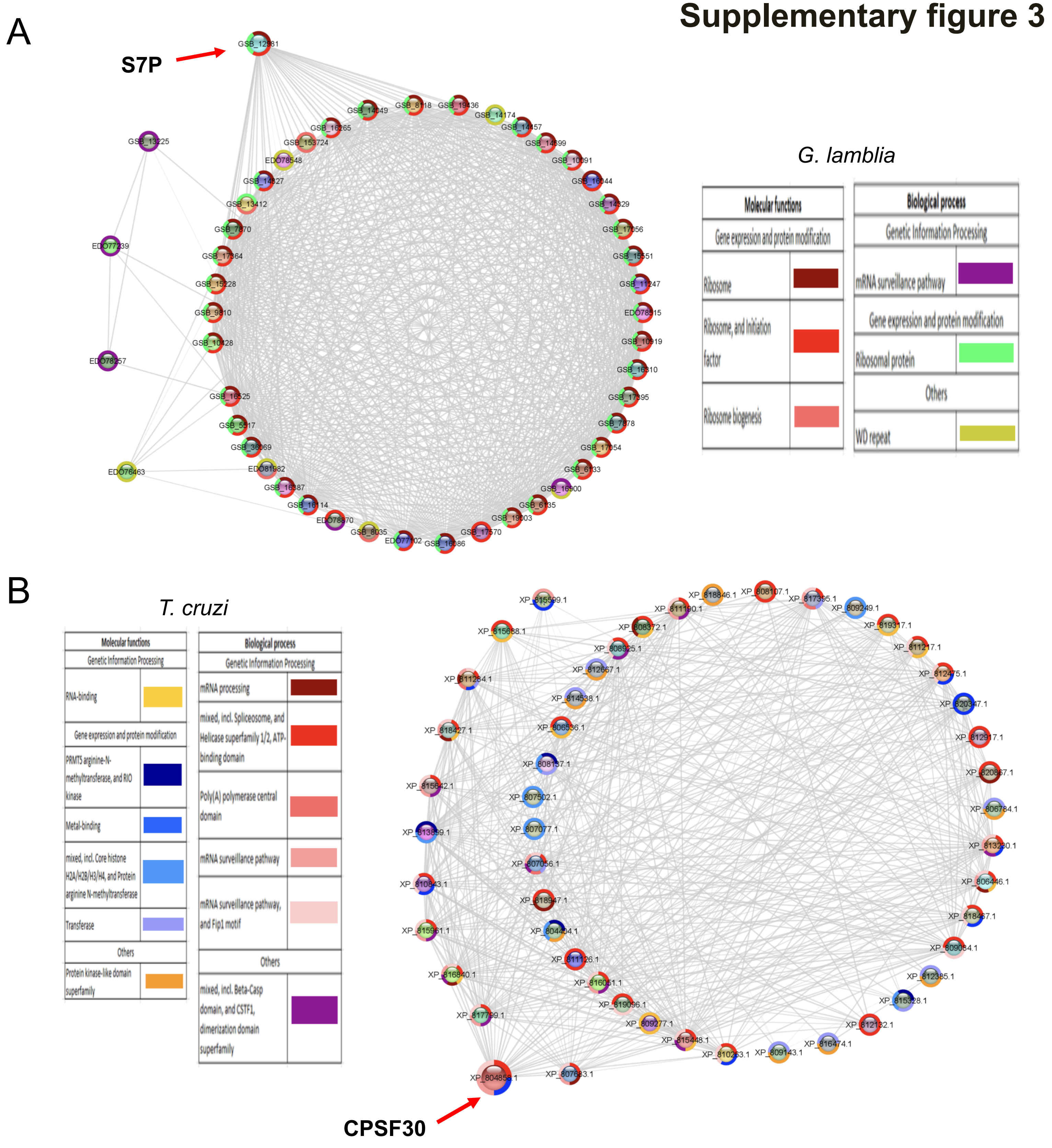

Supplementary figure 3. Analysis of polyadenylation factors interactions in other protozoan pathogens. Data were obtained from STRING database and EuPathDB Bioinformatics Resource Center with the corresponding parasite genome database including (A) *Giardia lamblia*, (B) *Trypanosoma cruzi*, (C) *Plasmodium berghei*, (D) *P. falciparum*, and (E) *Toxoplasma gondii*. The networks were visualized using the Cytoscape tool, principal proteins corresponding to polyadenylation factors were represented in the external network. Internal interactions represent proteins associate with polyadenylation process. Central hubs proteins are indicated with red arrows. GO terms including Molecular functions and biological process were carried out using KEGG Pathways, STRING clusters and Interior domains. Proteins were marked with the corresponding GO term color.

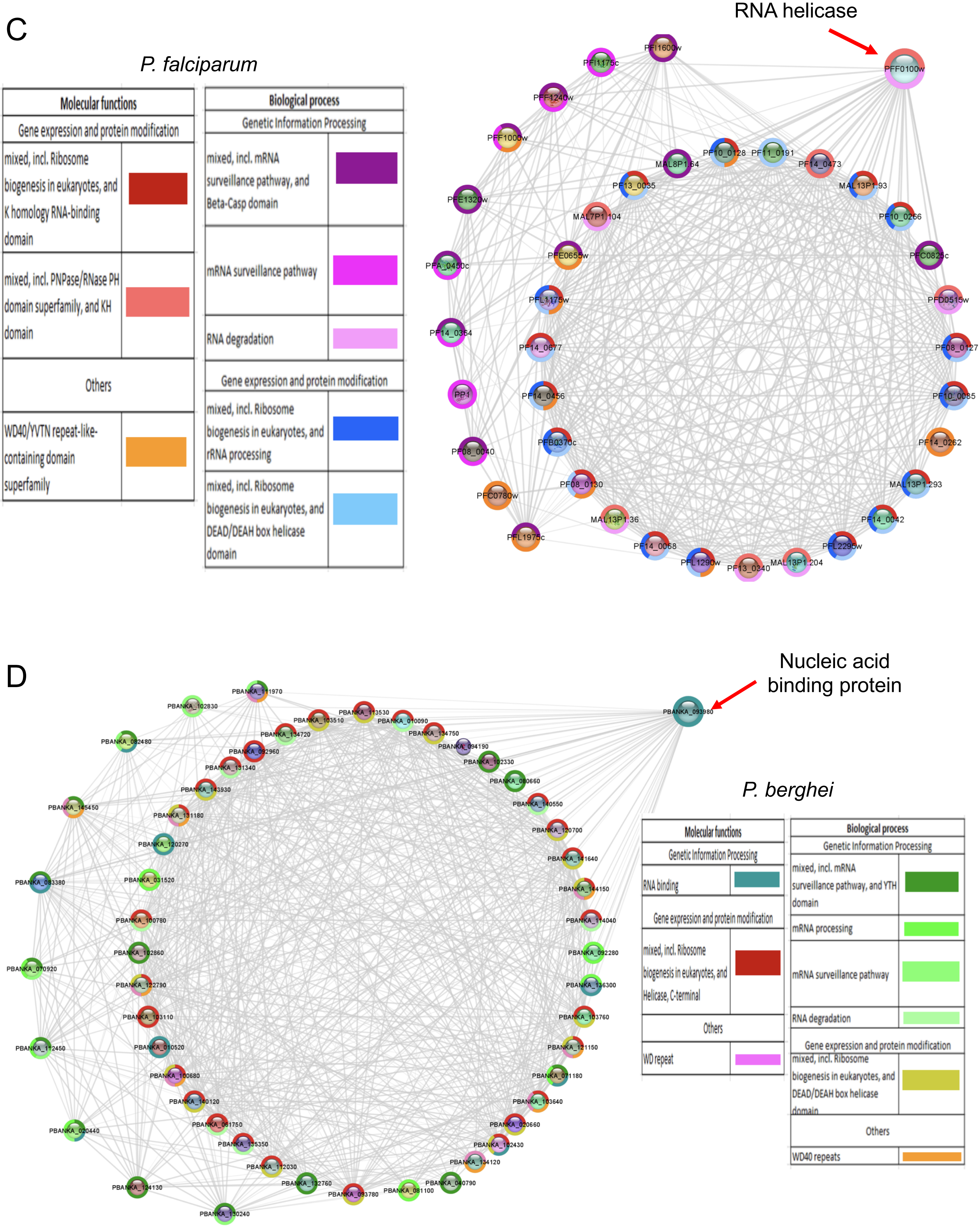

Supplementary figure 3. Part 3

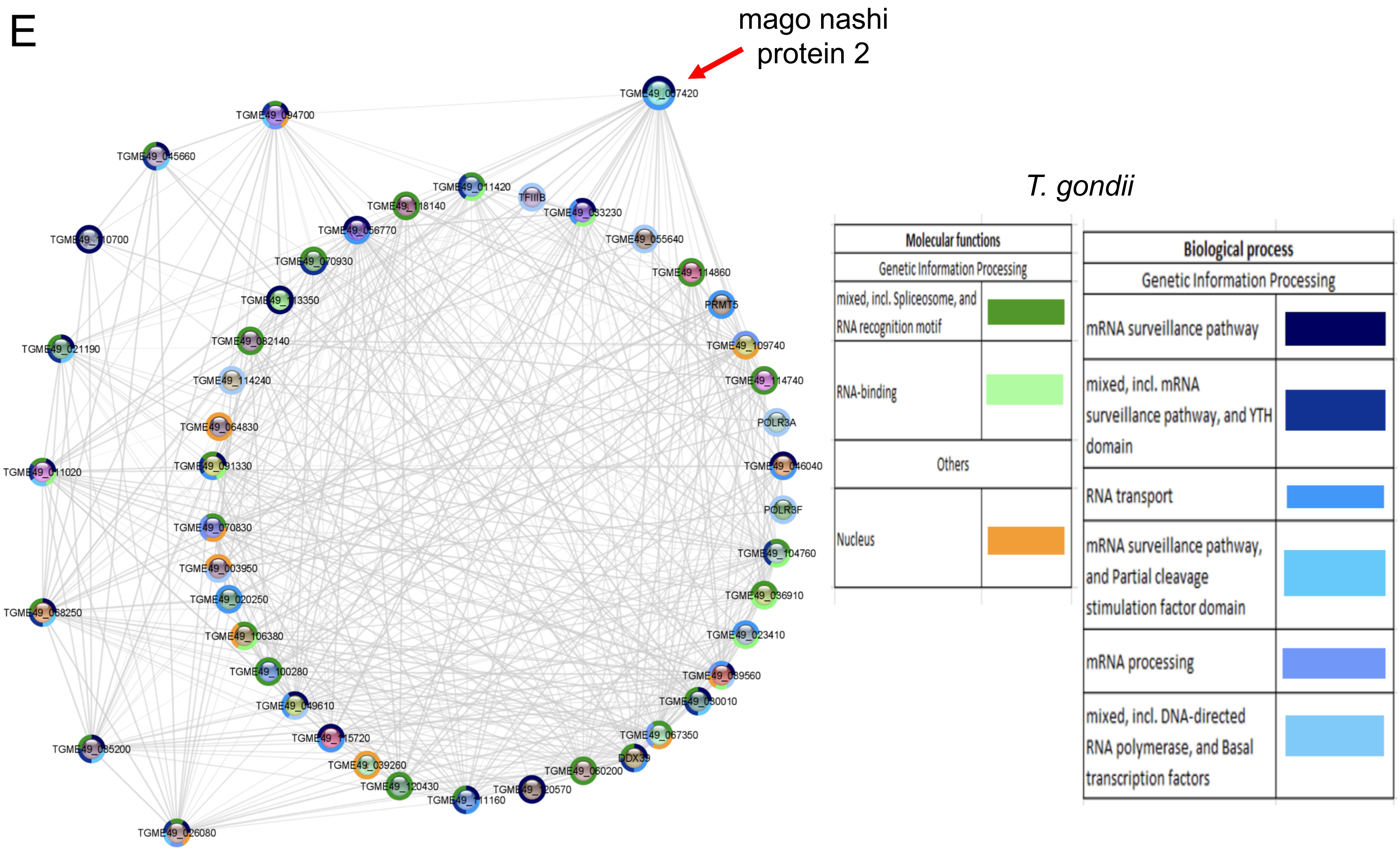

Supplementary figure 3. Part 3

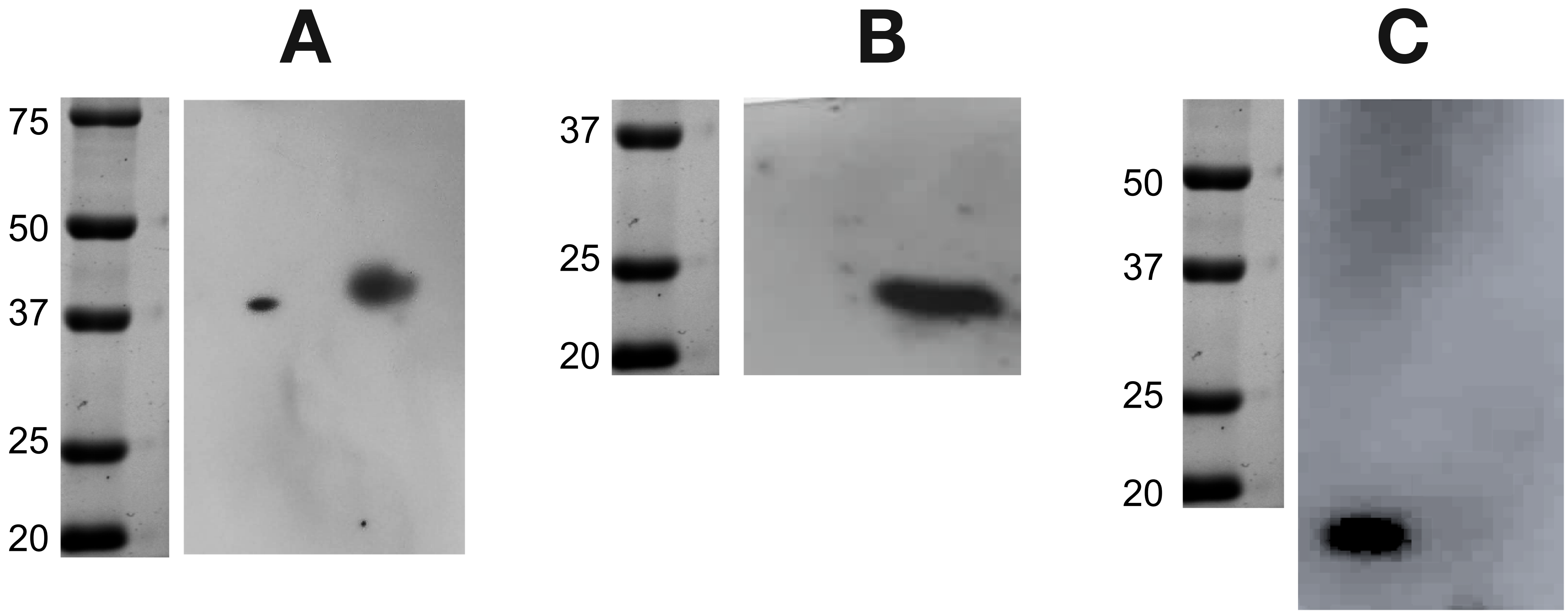

Uncropped figure of raw Western blot experiments shown in figure 8 using anti-HsCFIm25 (A), anti-EhPC4 (B) and anti-EhPSP (C) antibodies that confirm the recognition of parasite CFIm25 protein by heterologous antibody and the absence of cross contamination between CE (left) and NE (right).
